# Unraveling NK cell heterogeneity through single-cell sequencing: insights from physiological and tumor contexts for clinical applications

**DOI:** 10.3389/fimmu.2025.1612352

**Published:** 2025-07-21

**Authors:** Mingxin Shen, Yutong Liu, Liang Shao, Meng Qu, Shixin Song, Wei Sun, Hao Zhang

**Affiliations:** ^1^ Department of Thyroid Surgery, The First Affiliated Hospital of China Medical University, Shenyang, Liaoning, China; ^2^ Department of Obstetrics and Gynecology, Shengjing Hospital of China Medical University, Shenyang, China

**Keywords:** single-RNA sequencing, NK cell, heterogeneity, tumor microenvironment, drug intervention

## Abstract

Natural killer cells (NK cells) are important immune cells within the tumor microenvironment (TME) and are considered the first line of defense in tumor immunity. Although many studies have focused on the role of NK cells in tumor therapy, the heterogeneity of NK cells complicates the investigation of the complex mechanisms within the tumor microenvironment. Single-cell sequencing technology, with its high-resolution capability, reveals the gene expression profiles of individual NK cells, highlighting their heterogeneity and providing more accurate information for NK cell therapy. This article begins by addressing the mechanisms underlying the formation of NK cell heterogeneity, emphasizing the significance of differentiation, development, and tissue residency in establishing this heterogeneity. It also summarizes the advances in the study of NK cell heterogeneity under physiological conditions and in tumor environments using single-cell sequencing technology. Finally, it analyzes the dynamic changes of NK cells within the tumor microenvironment under various therapeutic approaches to explore drug effects and resistance mechanisms, as well as to optimize therapeutic options. Investigating the mechanisms of tumor progression and drug intervention at the single-cell level will provide new perspectives for personalized treatment strategies centered around NK cells.

## 1 Introduction

**Figure 1:**
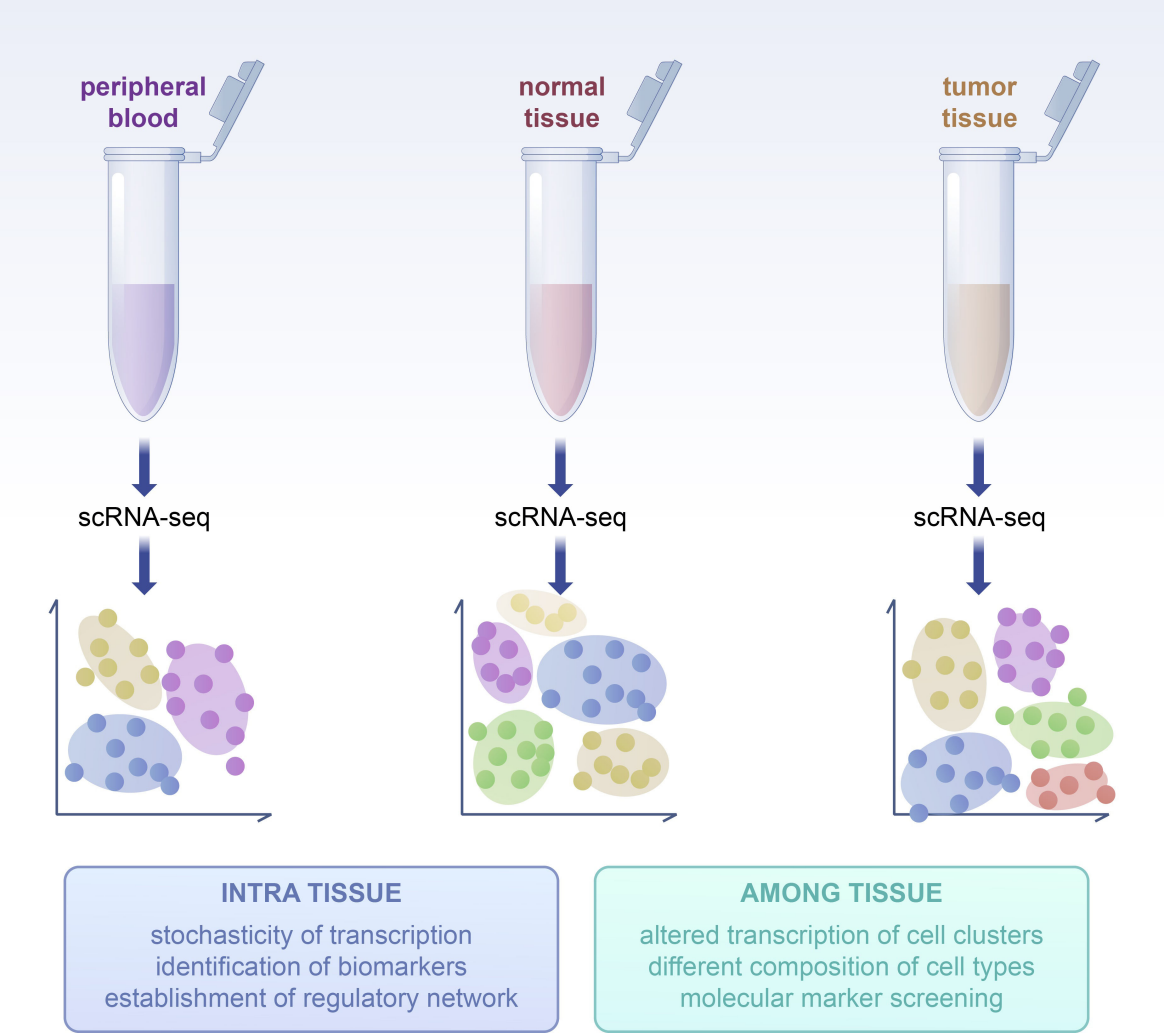
Basic process of scRNA-seq analysis. Including the following steps: 1. Dissociation of single cell 2. RNA hybridization 3. cDNA amplified by PCR 4. Library building 5. Sequencing 6. Bioinformatics analysis.

Single-cell sequencing technology enables the tracking of cellular development and differentiation, and by analyzing single-cell data from various developmental stages, it elucidates cellular differentiation pathways and fate decisions. A study that integrated single-cell RNA sequencing, antigen receptor sequencing, and spatial transcriptomics reconstructed the developmental trajectory of the human immune system and mapped its distribution across multiple organs in the body (1). Beyond the enumeration of clusters, single-cell transcriptomics has significantly enhanced our capability to characterize cell states from an individual cell perspective (2). This technology is instrumental in uncovering cellular heterogeneity and has emerged as a powerful tool for investigating cellular diversity within complex biological systems. The basic sequencing process is illustrated in the following figure (**Figure 1**). Through single-cell sequencing, researchers can identify the heterogeneity within cell populations and discern the variations in cell states and functions. For instance, Han X et al. employed single-cell mRNA sequencing to determine the cellular composition of major human organs and established the Human Cell Landscape (HCL) schema (3). With the advent of microfluidic technology and integrated sequencing strategies, the application of single-cell sequencing has significantly expanded, particularly in the field of oncology, where it holds substantial promise. Historically, researchers have often equated the uniqueness of individual cells with the characteristics of the population. However, recent advancements in oncology research have highlighted the substantial heterogeneity among tumor cells from different sources and even within individual tumors, underscoring the importance of single-cell analysis (4). Innovative single-cell genomics techniques and spatially multiplexed *in situ* methods now offer an unparalleled level of resolution to comprehend the intricate interactions within tumor ecosystems. A growing body of research is contributing to the development of tumor atlases, which are anticipated to profoundly influence our understanding of cancer biology and potentially enhance the detection, prevention, and treatment of cancer, leading to precision medicine for cancer patients and those at risk (5). Innovative single-cell genomics techniques and spatially multiplexed *in situ* methods now offer an unparalleled level of resolution to comprehend the intricate interactions within tumor ecosystems. Since single-cell sequencing technology was selected as the Method of the Year by Nature magazine in 2013 (6), it has been increasingly utilized in both tumorology basic science and clinical research, successively characterizing the TME in glioblastoma, melanoma, breast tumors, colorectal cancer, squamous cell carcinoma of the skin, and lung cancer (7–12).

**Figure 2:**
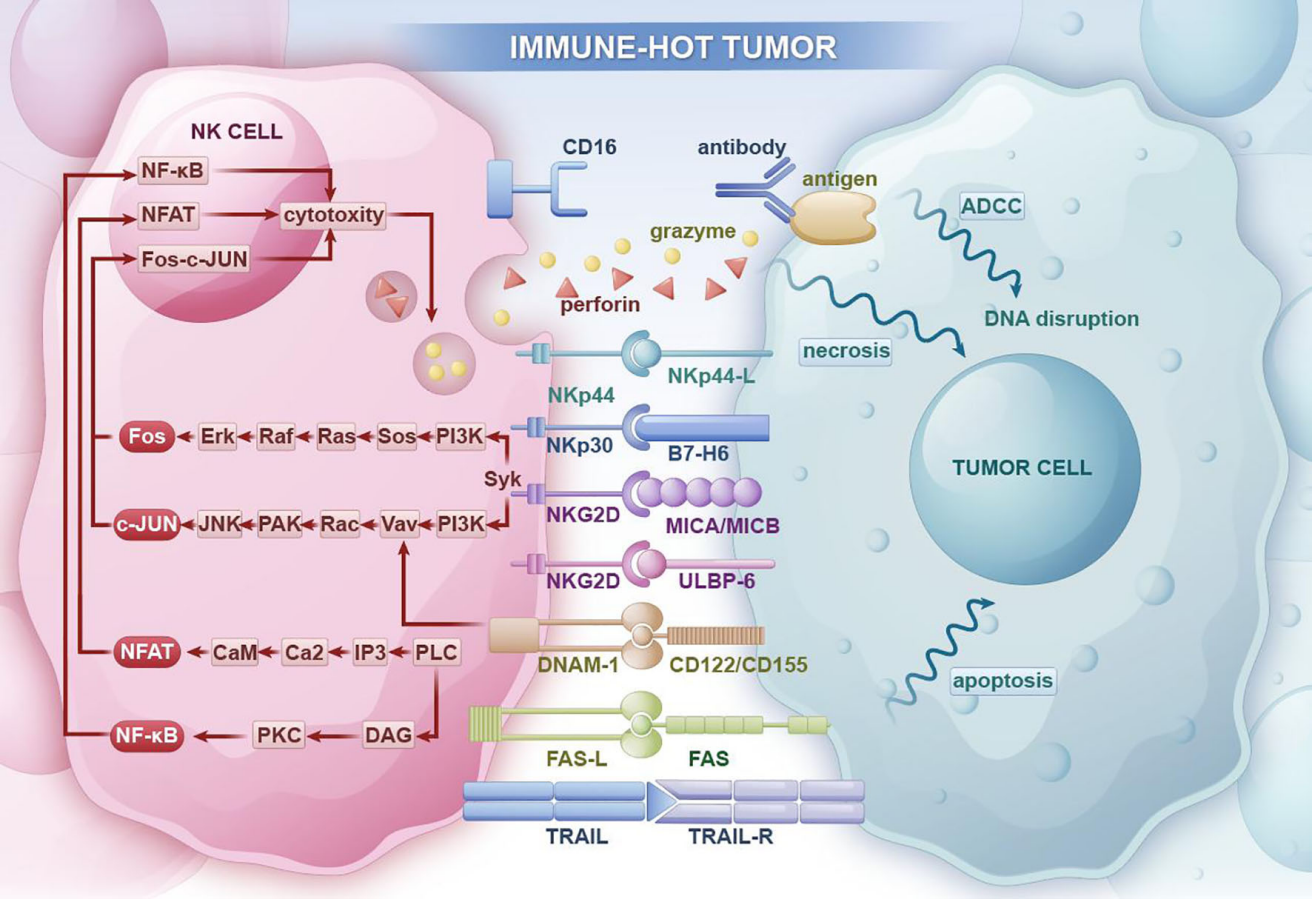
In immune-hot tumor regions, NK cells can promote tumor cell necrosis through antibody-dependent cellular cytotoxicity (ADCC) and the release of perforin and granzymes. They can also inhibit tumor occurrence and progression through apoptosis receptor ligands that induce tumor cell apoptosis pathways.

Natural killer (NK) cells were first identified in the 1970s through cytotoxicity assays conducted on cancer patients (13, 14). These cells demonstrated the capacity to lyse a diverse array of tumor cells. NK cells play a critical role in anti-tumor immunity and function as the primary line of defense in the immune response against tumors. (**Figure 2**) The killing mechanisms of NK cells are primarily categorized into two pathways. The first involves the secretion of cytoplasmic granule toxins, including perforin and granzymes, which activate cell death mechanisms. The second pathway leads to classical caspase-dependent apoptosis by the binding of death receptors, such as Fas/CD95, on target cells to their corresponding ligands, such as FasL, on NK cells, resulting in target cell killing (15, 16). NK cells are the first recognized subset of innate lymphoid cells (ILCs) and play a crucial role in the anti-tumor response of innate immunity (17), characterized by their autonomous ability to kill target cells and the high heterogeneity they exhibit within the tumor microenvironment (18).Recently, single-cell sequencing technology has proven instrumental in characterizing the heterogeneity and developmental processes of tumor-infiltrating T cells across various types of cancer (19). However, the understanding of NK cells remains incomplete. This review analyzes the sources of NK cell heterogeneity and provides a comprehensive overview of the development process of NK cells and the mechanisms of NK cell tissue residency to gain a deeper understanding of the core of tumor heterogeneity. It also explores advancements in the study of NK cell heterogeneity using single-cell sequencing technology in both physiological and tumor environments, particularly in solid tumors. Single-cell sequencing technology facilitates detailed subpopulation classification and functional studies of NK cells, constructs maps of the TME, and enhances research on intercellular interactions within the TME. Additionally, this review examines drugs and their interaction mechanisms, investigates the evolution of NK cells, and aims to establish efficacy evaluation models to study drug effects and resistance mechanisms.

## 2 Differentiation and tissue residency endow NK cells with heterogeneity

### 2.1 The differentiation and development as prerequisite of heterogeneity

NK cells are known to derive from CD34^+^ hematopoietic stem cells (20) and progress through several developmental stages, including precursor, mature, and effector phases, ultimately forming the common NK cell populations observed in peripheral blood. This developmental trajectory is characterized by cellular differentiation into distinct NK cell types, influenced by the microenvironment and the signals they receive. The differentiation and maturation processes of NK cells contribute significantly to the heterogeneity of NK cell populations. Typically, NK cells are categorized into CD56^dim^CD16^+^ and CD56^bright^CD16^-^ subsets based on the expression levels of CD56 and CD16. These subsets differ significantly in cytolytic activity, cytokine production, and proliferative capacity, and they fulfill unique roles in the immune response (21). CD56^dim^ NK cells, which constitute approximately 90% of peripheral blood NK cells, express high levels of Fcγ receptor III (FcγRIII, CD16) and killer cell immunoglobulin-like receptors (KIR) and contain abundant perforin and granzymes, conferring high cytotoxicity but low cytokine production. In contrast, CD56^bright^ NK cells have reduced quantities of perforin and granzymes, lower cytotoxicity, and a greater propensity for cytokine secretion, including IFN-γ, GM-CSF, and TNF-α (22, 23). This review initially approaches the formation of NK cell heterogeneity by examining the process of NK cell development, providing insights into the origins of NK cell heterogeneity.

#### 2.1.1 The traditional view of NK cell development process

**Figure 3:**
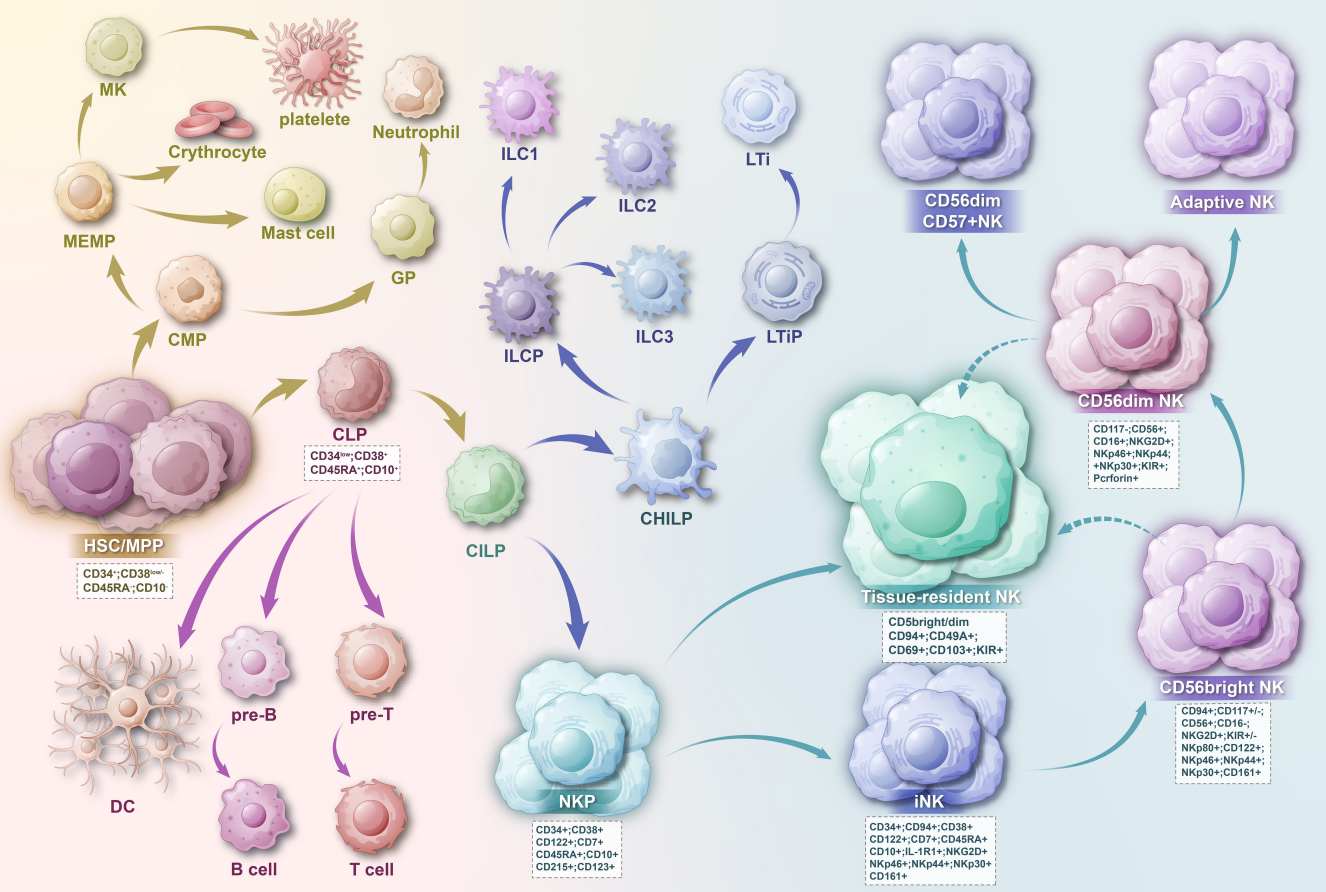
HSC cells develop into CMP and CLP, and the precursor of NK cells is the CHLP cell developed from CLP cell. CHLP cells then differentiate into CHILP cells, subsequently leading to the transition into ILC cells and NK cells. As NK cells approach maturity, they can migrate to tissues to form tissue-resident NK cells. The developmental outcome of NK cells is CD56dim CD57+ NK cells.

The linear model has traditionally been the prevailing perspective on NK cell development, and recent insights into ILC subsets have contributed to a more nuanced understanding of this developmental trajectory. (**Figure 3**) According to the linear model, hematopoietic stem cells (HSCs) differentiate into common lymphoid progenitors (CLPs). CLPs have the potential to commit to various ILC progenitor lineages, including B progenitor cells (pre-B), T progenitor cells (pre-T), NK progenitor cells (NKP), and others (24–26). CLPs subsequently differentiate through an intermediate stage of pre-NK progenitors (pre-NKPs) into NKPs (27, 28). The acquisition of the common β-chain of CD122 and IL-15 by NKPs represents a critical milestone in NK cell differentiation. The expression of CD122 signifies the irreversible commitment of NK cells from CLPs, while IL-15, produced by hematopoietic cells, monocytes, and dendritic cells, is thought to facilitate NK cell development in the bone marrow (29). Following this, NKP cells differentiate into immature NK (iNK) cells, which are characterized by increased expression of IL-1R1 and the emergence of CD314 (NKG2D), CD335 (NKp46), CD337 (NKp30), and CD161 (the human homolog of NK1.1 in mouse). The subsequent transitional stage is marked by the appearance of CD56^bright^ NK cells, which display high levels of CD56 and peak expression of NKG2D, NKp46, NKp30, and CD161. NK cells at this stage can be subdivided into phase 4a NK cells, which express more IL-22, and phase 4b NK cells, which express higher levels of transcription factors T-BET and EOMES and are capable of producing interferon-γ and thus cytotoxicity (30). CD56^bright^ NK cells eventually transition into CD56^dim^ NK cells, distinguished by increased CD16 expression, decreased CD56 expression, and the expression of various CD158 (KIR) subtypes (24, 26). CD56^dim^CD16^+^ NK cells can be further classified into subpopulations based on CD57 expression, including CD56^dim^CD16^+^ NK cells and CD56^dim^CD16^+^CD57^+^ NK cells, with the CD56^dim^CD16^+^CD57^+^ subset being considered the terminally differentiated subset, generated by prolonged stimulation from antigens or inflammatory signals (26, 31, 32). As the human NK cell developmental system has been refined, the developmental process of NK cells in mouse models has also been better characterized, aligning with this linear model. A recent study proposed a four-stage developmental model for the further progression of NKP cells according to the expression of NKp46. The NKP population sequentially matures into the CD122^+^NK1.1^−^CD49b^+^NKp46^−^ iNK-a population, the CD122^+^NK1.1^+^CD49b^−/+^NKp46^−^ iNK-b population, and finally, the CD122^+^NK1.1^+^CD49b^+^NKp46^+^ mNK population. These four NK cell populations display distinct phenotypic differences in cell surface markers, transcription factors, and effector molecules (33).

Notably, ILC cells share characteristic markers with NK cells, and the homology between them has been significantly enhanced through exploration, which has been pivotal in elucidating the developmental trajectory of NK cells and constructing the developmental lineage. It is recognized that the precursors of NK cells may diverge from those of ILC1s, ILC2s, and ILC3s. According to the development and functions of ILCs, including the production of cytokines and the expression profile of transcription factors, ILCs can be divided into five major subsets, including NK cells, ILC1s, ILC2s, ILC3s, and LTis (lymphoid tissue inducers) (34, 35). Common innate lymphoid progenitors (CILPs) are considered the intermediate stage between CLPs and the development of ILCs. CILPs can differentiate into NKPs or common helper innate lymphoid progenitors (CHILPs), which subsequently produce lymphoid tissue inducer progenitors (LTiPs) and innate lymphoid cell precursors (ILCPs). LTiPs differentiate into LTis, while ILCPs further differentiate into ILC1s, ILC2s, or ILC3s (34, 36), and NKP cells develop into mature NK cells along the well-known linear model. (**Figure 3**) However, this model cannot explain all experimental analytical conclusions, and many studies have supplemented this known developmental model, with many unknown directions yet to be explored. A study using global transcriptomics, flow cytometry analysis, and genetically engineered mouse models found that NK cells in the tumor microenvironment can depend on TGF-β to convert into intermediate ILC1 groups and ILC1 groups (37). Furthermore, emerging evidence suggests that previously identified ILCP cells retain the capacity to generate NK cells (38–41), indicating that the differentiation and commitment process within the ILC lineage is more intricate than previously thought. Additionally, Chen L et al. found that the CD34−CD117^+^ ILCP population can express CD56, produce NK cells and ILC3s, but not ILC2s. *In vitro* molecular and functional analysis and lineage differentiation analysis have proven that the final step of ILC2 development is independent and non-overlapping with NK cells and ILC3s (40). It is worth noting that NK cells and ILC1 together form group 1 ILCs, whose development and function rely on the T-box transcription factor T-bet and are characterized by the production of interferon-γ (IFN-γ) (34). An accurate understanding of the developmental relationships between NK cells and other cells within the ILC lineage, as well as their interactions, is essential for constructing a comprehensive NK cell developmental lineage.

Additionally, many questions remain unresolved. For example, whether there are still undefined subgroups in the development process of NK cells, from which stage NK cells separate from T/B cells and become independently developed individual cells, and the developmental process between ILCs and NK cells has not yet been precisely defined.

#### 2.1.2 Further dissection of NK cell development process under single-cell sequencing

Deciphering the cellular state transitions behind immune adaptation is fundamental to advancing biology. Through the application of single-cell sequencing, researchers are able to track the fate of individual cells, revealing the genetic changes as cells transition from a single state to new cell states and types, which helps to understand the development process of multicellular organisms. Although single-cell sequencing has not been extensively applied to the study of NK cell development, its research achievements are still encouraging, as it is expected to construct a complete developmental system from a single-cell perspective and to deeply understand the ontogeny of NK cells. Meanwhile, scRNA technology plays a key role in exploring ILC heterogeneity (35, 42), and it is expected to provide clues for the origin and understanding of NK cells and ILCs.

scRNA-seq technology can further elucidate the reliability of the linear model, explore the continuity of the developmental process, discover new subpopulations, and define the developmental process based on gene expression patterns. Yang et al. (43) used scRNA-seq technology to study the heterogeneity of NK cells in the peripheral blood and bone marrow of healthy individuals, and transcriptomic and pseudotime analysis confirmed the progression from CD56^bright^ NK to CD56^dim^ NK. A study on liver cancer also identified the continuous process of transformation from CD56^bright^ NK cells to CD56^dim^ NK cells through single-cell analysis (44). Furthermore, Yang et al. (43) identified a transitional NK cell population that bridges CD56^bright^ NK cells and CD56^dim^ NK cells, characterized by intermediate expression of marker genes associated with either CD56^bright^ or CD56^dim^CD57^+^ NK cells. More recently, Melsen JE et al. (45) discovered the CD56^dim^GZMK^+^ subpopulation, which is considered an intermediate stage between CD56^bright^ and CD56^dim^ NK cells. This subpopulation exhibits shared characteristics with both CD56^bright^ and CD56^dim^GZMK^-^ NK cells and displays higher expression of SELL (CD62L) and lower expression of GZMB, PRF1, FCGR3A (CD16), and FGFBP2 in comparison to CD56^dim^GZMK^-^ cells.

Furthermore, single-cell sequencing has provided insights into the point of divergence of NK cells from the lymphoid lineage. A study on the single-cell transcriptomic atlas of human blood cells revealed through pseudotime analysis that memory B cells and NK cells are distributed on two different branches, which better reflects the distinct trajectories of differentiation between B cells and NK cell populations. They also found that NK cells and T cells lack continuous transcriptional transitions from progenitors to differentiated cells and inferred that these cells may deviate from T cell development during the macro-DN(Q) stage of gene expression acquisition (46). Another study, utilizing single-cell RNA sequencing and adaptive immune receptor (AIR) sequencing (scVDJ-seq), has employed TRBJ frequency to create a V(D)J feature space, as all T, ILC, and NK cells express TRBJ. The inferred trajectories suggest that ILC/NK cells diverge from T cell development at the DN (early) and DN (Q) stages, while also indicating that T cells and ILC/NK cells may share a common initial developmental stage before diverging prior to the production of productive TRB, TRG, and TRD (47). This provides better evidence for the separation of NK cells from the lymphoid lineage. Meanwhile, scRNA-seq research reveals a more continuous transcriptional differentiation landscape, although lacking classical defined precursor stages, it contributes to a more systematic understanding of NK cell development (48–50).

Notably, the relationship between the ILC and NK cell developmental lineages and the heterogeneity between them has been better authenticated under single-cell sequencing, and these studies are expected to provide clues for the origin and understanding of NK cells and ILCs. A study identified common ILC1/NK cell progenitors and referred to them as aceNKPs. Single-cell RNA sequencing of the descendants of aceNKPs in the lung confirmed that aceNKPs are not only progenitors of NK cells but also of ILC1 cells. It was found that the descendants of aceNKPs could not be separated according to the traditional dichotomy between ILC1 and NK cells, and no clear demarcation of the transcriptional profiles between the ILC1 and NK cell lineages was observed, indicating that these closely related group 1 innate lymphocytes are indistinguishable. Furthermore, a single-cell study on tissue-resident NK cells (trNK) in the skin found that trNK cells retained the expression of the transcription factor ectodermal protein EOMES and did not acquire Hobit expression, indicating that trNK cells do not transition into ILC1, and ILC1 do not convert into trNK cells (51), challenging the notion that NK cells can readily convert into ILC1. Moreover, the heterogeneity between NK cells and ILCs has been a hot topic in recent years, and a comprehensive understanding of this heterogeneity is crucial for studying the origin of both and for the identification and selection of cells in single-cell sequencing. As note, ILC cells share typical markers with NK cells, and in humans, NK cells (c-kit^-^) are typically distinguished from ILC3 (c-kit^+^) (52). In mice, the distinction between NK cells and ILC1s is often based on the expression of EOMES, with NK cells (CD49a^-^ CD49b^+^) and ILC1s (CD49a^+^ CD49b^-^) being differentiated by the differential expression of integrins CD49a and CD49b (53). Lopes et al. (35) used single-cell RNA sequencing and CITE-seq to explore the heterogeneity between inter-tissue NK and ILC1 and defined syndecan-4 as a marker to distinguish mouse ILC1 from NK cells. They also found that the expression of EOMES, GZMA, IRF8, JAK1, NKG7, PLEK, PRF1, and ZEB2 defines NK cells, while IL7R, LTB, and RGS1 distinguish ILC1s from NK cells in both mice and humans. McFarland et al. (42) employed scRNA sequencing to clarify the gene signatures of mouse ILC1^-^NK cells obtained from tissue, tumors, and circulation, identifying unique phenotypic markers, transcription factors, and metabolic characteristics that distinguish tissue NK cells from circulating NK cells and ILC1. While these studies provide important clues for the investigation of NK cell development, it is acknowledged that the process of NK cells and ILCs developing from progenitors has not been thoroughly investigated. Therefore, it is imperative to further deepen our understanding of the origin of NK cells, as this knowledge will inform ongoing research into NK cell heterogeneity.

### 2.2 Tissue residency as a core factor in NK cell heterogeneity

Most of our knowledge of NK cells comes from studies on mouse spleen, bone marrow, and human peripheral blood mononuclear cells (PBMC). These cells are commonly referred to as conventional NK cells (cNK). In recent years, a distinct subset of NK cells, known as tissue-resident NK cells, has been identified and has garnered significant interest. Human NK cells typically comprise 5-15% of peripheral blood lymphocytes and are abundant in bone marrow (BM), liver, uterus, spleen, and lungs, with a smaller population also found in secondary lymphoid tissues (SLT) and mucosal-associated lymphoid tissue (MALT) (54–56). In 2016, Lughart G et al. identified a CD69^+^CXCR6^+^ NK cell subset in lymphoid tissues, which is considered an independent NK cell population with distinct phenotypes and functions (57). Unlike cNK, trNK are exclusively found in peripheral tissues. While there is phenotypic similarity between peripheral blood NK cells and trNK cells, unique transcriptional profiles indicate that the cellular environment is a key factor in NK cell heterogeneity within the same developmental stage (58). By tracing the origin of trNK cells and their unique developmental process, we can further investigate the diversity of NK cell expression and subpopulation ratios influenced by the tissue, as well as characterize the heterogeneity of tissue-resident NK cells under physiological conditions. It is important to note that previous studies have primarily focused on the heterogeneity of tumor NK cells, often overlooking the diversity of tissue cells under physiological conditions—a distinction that should not be disregarded. NK cells in the TME originate from various sources, including cNK and trNK. A comprehensive understanding of NK cell heterogeneity under physiological conditions is crucial for analyzing the differences between NK cells in the TME and non-diseased tissues, providing a significant reference for personalized immunotherapy strategies for tumors.

#### 2.2.1 The unique generation process of tissue-resident NK cells contributes to the heterogeneity of NK cells

Previous studies have indicated that during differentiation, both immature and mature NK cells migrate from bone marrow parenchyma to sinusoids and subsequently enter the bloodstream (59, 60). The circulation of blood between lymphoid and non-lymphoid organs facilitates their entry into secondary lymphoid tissues and various other tissues at different stages of development, differentiation, and activation (54, 61, 62). Some specialized subsets are capable of returning to specific niches within the BM to fulfill functions such as control and surveillance of malignant cells (59). However, more detailed research has revealed the presence of trNK cell populations in certain tissues, which are believed to establish residency early in life without the need for microbial stimulation during development and are capable of self-maintenance (63). Moreover, due to the functional and residential similarities between ILC1 and NK cells, ILC1 found in organs has traditionally been considered a subtype of trNK cells (64). Despite sharing overlapping transcriptomic regions and dependence on IL-15, the developmental pathways of these two cell types are quite distinct (36). NK cells derive strictly from NKP cells, which are committed to producing NK cells and do not generate other ILC subsets. It is evident that the trNK cell population exhibits distinct developmental requirements from cNK cells and ILC1 and is considered unique in ontogeny (65).

Accurate knowledge of the origin of tissue-resident NK cells is crucial for understanding the roles of NK cells in various tissues and their physiological functions, and it forms the basis for further insights into the functions of NK cells under pathological conditions such as inflammation and tumors. Studies have suggested that trNK cells may originate from tissue-resident progenitor cells rather than following the bone marrow-bloodstream pathway. It has been observed that NK progenitor and immature cells mature in secondary lymphoid tissues such as spleen, lymph nodes, and tonsils (24, 25, 66). These progenitor and immature NK cells can also be inoculated into the lymph nodes and intestine, where they reside and act as reservoirs for the maintenance of NK cell populations (55). Furthermore, a study utilizing 12-color flow cytometry identified pre-NKPs, which represents an intermediate developmental stage between the upstream CLP cells and the downstream NKP cells and was previously considered the initial stage of commitment to the NK lineage. Unlike other transplant recipients, intrathymic injection of these progenitors did not produce NK cells, suggesting that thymic NK cells have a distinct developmental origin (28). Some studies have also provided evidence that DN1 thymic progenitor cells can differentiate into thymic NK cells, which are distinct from cNK cells (67). These findings indicate the presence of NK cell progenitors in the human thymus that can differentiate and develop independently to form tissue-resident NK cells within the thymus. Additionally, in mouse lymph nodes, certain NK cell subsets are thought to originate from NKP cells in the thymus, while others are thymus-independent NKP cells originated from lymph nodes, indicating the presence of NK progenitors in lymph nodes (68). The advent of single-cell sequencing technology, with its high-resolution capabilities, has further characterized the developmental process, providing compelling evidence for the existence of trNK progenitor cells. Recent studies combining single-cell transcriptomics, flow cytometry, and TCRD rearrangement data have demonstrated that non-T cell lineages, including NK cells, can develop in the human thymus (69), confirming the presence of NK cell progenitors in tissues capable of developing into trNK cells. In 2018, Eric Vivier et al. identified four subgroups in the human spleen at single-cell resolution, among which hNK_Sp3 and hNK_Sp4 cells did not resemble any blood NK subgroups and appeared to be spleen-specific (70). Three years later, they used the same technology for single-cell sequencing of the bone marrow and found that spleen NK 0 (hNK_Sp3) cells could also differentiate into spleen CD56^dim^ NK cells and CD56^bright^ NK cells. This contradicts the notion that NK cells develop exclusively in the bone marrow, suggesting that some precursors of NK cells are enriched in extramedullary tissues (71). Moreover, researchers have identified CD117^+^ ILC clusters lacking mature ILC markers in the colon and lung through single-cell sequencing, which, unlike circulating ILCs, exhibit a transcriptome consistent with tissue residency and may represent resident precursors of mature ILCs in these tissues (35). These studies confirm that the development of NK cells not only originates from the bone marrow but also involves some extramedullary tissues that harbor progenitors of the NK cell lineage, which differentiate into trNK cells with a distinct developmental process from cNK cells and present unique growth requirements.

Notably, in specific situations such as infection and tumors, some studies have also observed that cNK cells can be recruited to tissues to fulfill various roles. Recent investigations have revealed that cNK cells are redistributed and persistently located at previously virus- and bacteria-infected skin sites through a mechanism that promotes tissue retention, and they differentiate into TCF1^hi^CD69^hi^ NK cells that are transcriptionally similar to CD56^bright^TCF1^hi^ NK cells in human tissue (51, 65). Utilizing single-cell sequencing technology, Dean I et al. (72) discovered in mouse solid tumors that cNK cells recruited from the circulation can transform into CD49a^+^ NK cells that adapt to the tumor environment, establishing a tumor-resident state and expressing markers of tissue residency such as CD49a and CD69. These cells also exhibit effector functions, including the killing of cancer cells and the promotion of the recruitment and activation of dendritic cells (DCs). Another study, employing scRNA-seq, found that circulating NK cells are recruited to the salivary gland in a CX3CR1^-^dependent manner, where they develop into NKRM (tissue-resident memory-like NK) cells. These long-lived tissue-resident cells prevent autoimmunity by eliminating CD4^+^ T cells in a TRAIL-dependent manner, providing sentinel protection for the host and playing a crucial role in maintaining immune homeostasis, limiting immune responses, and preventing autoimmune damage (73).

By elucidating the origin and developmental pathways of trNK cells, we can ascertain that the tissue-resident nature of these cells may arise from pre-existing tissue-resident NK cell precursors within the tissue, thereby becoming a core element of NK cell heterogeneity. Concurrently, our research offers researchers valuable insights. For instance, in recent years, the application of NK cell therapy has expanded significantly. Beyond the challenge of poor targeting affecting NK cell efficacy, insufficient infiltration of NK cells into target tissues remains a substantial obstacle that must be addressed. Our research opens up new directions for researchers, as NK cell precursors can be used as research objects to regulate the tissue-resident capacity and infiltration level.

#### 2.2.2 Tissue influences NK cell differentiation leading to heterogeneity

As previously discussed, growing evidence supports the notion that the tissue microenvironment influences the expression profile of NK cells. We can discern the role of the tissue environment in the development of trNK cells by comparing the environmental differences between trNK cells and cNK cells, as well as by examining the variations in trNK cells across different tissues.

Tissue-resident trNK cells and conventional cNK cells may originate from distinct precursor cells and exhibit significant disparities in their differentiation, development, and transcriptional profiles. Unique transcriptional signatures indicate that the cellular environment is a key factor in generating NK cell heterogeneity within the same developmental stage (58, 74, 75). A consistent feature among tissue-resident NK cells across various tissues is their expression of tissue-resident markers and a less mature phenotype compared to cNK cells, with their development being less reliant on the transcription factor NFIL3 (76). In both trNK cells and cNK cells, the distinct cytokine milieus influence their activation profiles. IL-21R is highly expressed in trNK cells, while IL-18R is predominant in cNK cells, and their respective ligands, IL-21 and IL-18, primarily facilitate the activation of trNK and cNK (77). Notably, cNK cells are absent in NFIL3-deficient mice, whereas trNK cells persist in the liver, skin, and uterus of these mice, suggesting differential requirements for transcription factors during development (78). The different tissue environments also result in distinct cytokine expression profiles between trNK cells and cNK cells. For instance, liver trNK cells exhibit higher expression of TNF-α and GM-CSF compared to cNK cells (78). A recent study on liver cancer demonstrated that the local immunosuppressive microenvironment during the chronic liver injury phase affects peripheral NK cells but does not impact tissue-resident NK cell subsets (79). This suggests that different NK cell types exhibit varying tolerances to the immune microenvironment.

Additionally, significant variations exist among NK cells across different tissues, which are evident in the differential distribution of NK cell subsets, as well as in the disparities in phenotype, function, and transcription factor requirements of NK cells in various tissues. These differences reflect the impact of the local microenvironment on the acquisition of tissue-specific traits by trNK cells (76). Studies have demonstrated that the abundance of NK cells varies across different tissues. The prevalence of CD56^dim^CD16^+^ NK cells expressing CD57, a marker of terminal differentiation, differs between locations, with higher frequencies observed in tissues rich in CD56^dim^CD16^+^ NK cells such as blood, spleen, bone marrow and lung, and lower frequencies in locations like lymph nodes and intestines (55). In humans, CD56^bright^CD16− NK cells exhibit tissue-resident traits in multiple locations, while CD56^dim^CD16^+^ NK cells display characteristics of circulating cells in blood, spleen and bone marrow but can adopt a resident phenotype in lymph nodes and mucosal tissues. This distribution suggests that NK cells play a role in controlling viral infection and tumor progression in tissues accessible to circulation, while intestinal and secondary lymphoid sites may be less conducive to NK cell-mediated immune surveillance (55). Moreover, the expression profile of NK cells differs significantly across tissues. A recent study on mouse utilizing scRNA-seq and flow cytometry identified gene expression patterns in NK1.1^+^NKp46^+^ cells across various tissues, revealing substantial differences in the proportion of marker-positive cells in blood, spleen, inguinal lymph nodes, CD49α− liver, intestinal epithelial lymphocytes, intestinal lamina propria lymphocytes, liver, salivary gland, and uterus (42). In blood and other tissue sites, there is a bias towards the expression of GZMB in CD56^dim^ cells. Compared to CD56^dim^ NK cells in lymph nodes and intestines, the frequency of GZMB^+^ in CD56^dim^ NK cells in the lungs is significantly higher (55). Similarly, the transcriptional characteristics, phenotype, and function of ILCs are inherently influenced by their tissue microenvironment (80). Furthermore, the expression of transcription factors in trNK cells varies between species. For instance, trNK cells in livers of human and pigs express high levels of EOMES and low levels of TBox transcription factor 21 (TBX21), while trNK cells in mouse livers express high levels of TBX21 and low levels of EOMES (81). Tissue-resident NK cells exhibit significant differences in gene expression compared to conventional NK cells, and there is also substantial heterogeneity in receptor expression. (**Table 1**) These findings highlight the role of the tissue microenvironment in shaping the distinct expressions and heterogeneity of NK cells.

**Table 1:** The differential gene expression and receptor heterogeneity between tissue-resident NK cells and conventional NK cells under single-cell sequencing technology.

Organ	Tissue-resident NK cells	Conventional NK cells	Gene expression or receptor function	Function	Reference
liver,peripheral blood	CD 56 bright ihNK cells	CD 56 bright pbNK cells	CD56 bright ihNK cells:high expression of CD69,CD 38,CXCR6,CCR5,EOMES,FCER1G,IFNG,IL-2RB,IRF8,KLRB 1,RORA,S1PR5,STAT4,TGFBR2,TIGIT and ZBTB16;high levels of the co-inhibitory receptor TIGIT and decreased expression of T-bet (TBX 21) CD56 bright pbNK cells:increased expression of CXCR3, IL7R, ITGAM, S1PR1, and SERPINB1;high levels of DNAM-1	CD56 bright ihNK cells: regulate immune functions, exhibit anti-inflammatory activity, promote tissue residency and support cell proliferation CD56 bright pbNK cells: Involved in NK cell migration and development	(61)
liver,peripheral blood	liver trNK cells	GZMB+ cNK cells	increased expression of core genes KLRB1,GZMK,CD160,CXCR6 and EOMES	CXCR6+CD16− lrNK cells exhibit a greater capacity to produce IFN-γ and CD107a in response to IL-12/IL-18 stimulation *in vitro* increased liver infiltration when suffering from HBV infection	(82)
liver,peripheral blood	trNK cells	cNK cells	high expression of EOMES,FCER1G,TYROBP,CXCR6,GZMK,FGL2,IRF8,CCL4 and proliferation markers STMN1,HMGB2 and TUBA1B	FGL2 regulates the defense response against viruses IRF8 participates in the cellular response to interferon-γ CCL4 positively regulate the process of chemotaxis Proliferation markers possess self-renewal and proliferative capabilities	(81)
liver,peripheral blood	CD 56 bright NK cells in liver	CD 56 bright NK cells in peripheral blood	high expression of Eomes and transcription factor PLZF high proportion of cells expressing CXCR6 and CD69 low expression of T-bet	promote residency of NK cells in liver	(70)
spleen,peripheral blood	hNK_Sp1,hNK_Sp2,hNK_Sp3,hNK_Sp4	hNK_Bl1 and hNK_Bl2	increased expression of genes NFKBIA,TSC22D3,ANXA1 and genes encoding anti-apoptotic proteins BCL2A1 and MCL1 and genes encoding secretory proteins CCL3,XCL1,IFNG and GZMK, pro-inflammatory cytokine IL-32, and cell membrane proteins CD69,CD94,ICAM1 and CD161	function of defense, response to stimulation, response to cytokines, response to stress and signal transduction	(83)
spleen,peripheral blood	mNK_Sp1,mNK_Sp2,mNK_Sp3	mNK_Bl1 and mNK_Bl2	The transcriptomic characteristics between mNK_Bl1 and mNK_Sp1 cells, as well as between mNK_Bl2 and mNK_Sp2 cells show similarities, while the mNK_Sp3 subpopulation is absent in the blood.	—	(83)
bone marrow,peripheral blood	7 bone marrow derived clusters: CD56brightNK Transitional NK Active NK Adaptive NK Mature NK Terminal NK Inflamed NK	5 blood-derived clusters: CD56bright NK Transitional NK Active NK Mature NK Terminal NK	BM and blood exhibit similar NK cell heterogeneity, but different composition	—	(84)

## 3 Advances in the study of NK cell heterogeneity through single-cell sequencing

### 3.1 Advances in the study of NK cell heterogeneity in physiological conditions through scRNA-seq technology

NK cells’ capacity to recognize and eliminate malignant or infected cells through germline-encoded and nonclonal receptors offers clinical promise. However, this straightforward paradigm is complicated by the considerable heterogeneity observed within NK cell populations. Extensive research has been dedicated to understanding the heterogeneity of trNK cells (54, 61). Single-cell sequencing technology, particularly single-cell transcriptomics, offers an unbiased approach to elucidating cellular heterogeneity and cell states (82), thereby providing the technical framework necessary for identifying cell subpopulations and for gaining a deeper understanding of the heterogeneity of trNK cells both between and within different tissues. In this review, we summarize the recent studies on NK cell heterogeneity facilitated by single-cell sequencing, which contributes to a more profound understanding of the impact of tissue microenvironments on NK cell expression and the adaptive changes of NK cells. This knowledge enhances our grasp of the role of NK cell distribution and expression in physiological functions and establishes a foundation for investigating NK cell heterogeneity in the tumor environment.

In 2018, Adeline Crinier et al. conducted scRNA sequencing on spleens and peripheral blood of humans and mice, respectively, and compared the results. They identified hNK_Sp3 and hNK_Sp4 in human spleen and mNK_Sp3 in mouse spleen, which were not present in peripheral blood. These cells exhibited high expression of anti-apoptotic proteins and pro-inflammatory cytokines, indicating that tissue-resident NK cells possess enhanced defense responses, sensitivity to stimuli, cytokine responsiveness, stress tolerance, and signal transduction capabilities, all of which are associated with high NK functional activity (70). Another study revealed that compared to peripheral blood, bone marrow NK cell-derived clusters exhibited two additional derived clusters, termed “adaptive NK cells” and “inflammatory NK cells”, suggesting a functional diversity and inter-group heterogeneity (43). In 2020, Zhao J et al. (83) demonstrated that liver-resident NK cells exhibited increased expression of core genes KLRB1, GZMK, CD160, GZMK, and CXCR6 compared to GZMB^+^ cNK cells and displayed a greater capacity to produce CD107a and IFN-γ in response to IL-12/IL-18 stimulation *in vitro*, as well as enhanced infiltration in HBV-infected livers. In 2023, a single-cell sequencing study on pig liver revealed that trNK cells, compared to peripheral blood, showed increased expression of EOMES, FCER1G, TYROBP, GZMK, and CXCR6, as well as high expression levels of fibrinogen-like 2 (FGL2), interferon regulatory factor 8 (IRF8), and CCL4, which are involved in positive regulation of the defense response to viruses, the response to IFN-γ, and chemotaxis. Additionally, they also highly expressed proliferation markers HMGB2, STMN1, and TUBA1B, indicating that these cells possess proliferation and self-renewal capabilities (81).

At the same time, researchers have further classified NK cells based on their expression patterns, further exploring the heterogeneity of different NK cell subpopulations within tissues. Smith SL et al. (84) utilized scRNA-seq to identify seven blood NK subgroups (cluster0.1.2.3.4.6.8), each displaying distinct expression patterns of cytokines and receptors. These subgroups were categorized based on gene expression as CD56^dim^ NK cells, terminally differentiated CD57^+^ NK cells, CD56^bright^ NK cells, CD56^-^CD16^+^CD7^+^ NK cells, CIML NK cells, and cells undergoing senescence or death. The researchers also divided bone marrow NK cells into seven bone marrow-derived clusters based on their expression patterns and further investigated their respective functions. Among these, “inflammatory NK cells” respond to interferon stimulation and promote positive regulation of the inflammatory response; “activated NK cells” respond rapidly to stimulation; “adaptive NK cells” can enhance the positive regulation of gene sets involved in endoplasmic reticulum-to-Golgi transport vesicle formation and IFN-γ production, while “mature NK cells” exhibit potent cytotoxic functions 43. Moreover, the proportion and function of NK cell subpopulations can vary under different physiological conditions. Studies have shown that the proportion of NK/ILC1 cells in the uterus of pregnant mice is higher than that in non-pregnant mice, and the expression of GZMB, PRF1 and SPP1 is increased in the trNK#3 and Mki67^+^ trNK#3 subgroups, which is associated with cell lysis, endoplasmic reticulum stress response, and regulation of tissue remodeling (85). Notably, new subpopulations also emerge following HIV infection (86).

The study of NK cell heterogeneity under physiological conditions has delineated the differences between trNK cells and cNK cells, and it has detailed the diversity of NK cell subpopulations within various tissues. (**Table 2**) This research also suggests that when examining NK cell heterogeneity in the tumor microenvironment, it is crucial to distinguish between the differences between tumors and peripheral blood, as well as between tumors and unaffected normal tissues, thereby avoiding a simplistic view of the tumor microenvironment and fostering a more comprehensive, multi-dimensional understanding of these differences. In recent years, single-cell sequencing has primarily focused on the liver, spleen, uterus, and bone marrow, while organs such as the lung, thymus, lymph nodes, tonsils, kidney, and adipose tissue have been less explored. Previous studies have analyzed the heterogeneity of NK cells in these organs (57, 75, 93), and single-cell sequencing technology is expected to provide a more comprehensive and deeper understanding in the future.

**Table 2:** The classification of NK cell subpopulations and their corresponding functions in various tissues under non-tumor conditions using single-cell sequencing technology.

Origin of specimen	Organs	Subpopulation of NK cells	Gene expression or receptor function	Function	Reference
mice	liver,peripheral blood	cluster0-2	cluster 1 and 2: differential expression of NK cell receptor transcripts and cytolytic molecules, with Klrg1 being enriched in cluster 2. cluster 0 (cNK): high expression of GZMA, ITGA1, CD93, IL2RA, and IL18R1.	—	(87)
mice	spleen	cluster0-4	cluster 0 (cNK): high expression of TBX21,adapter molecules of activating receptors TYROBP and FCER1G, and transcripts of cytotoxic granule components GZMA,GZB3,NKG7,and PRF1	—	(87)
mice	uterus	cNK cells,trNKp cells,trNK1 cells,trNK2 cells	cNK cells: strongly express PRF1 and KLRB1A, and upregulate NK cell-mediated cytotoxic pathways trNKp cells: exhibit high expression of CDK1 and CDC20 trNK1 cells: elevated levels of LGALS3 expression trNK2 cells: enriched in Th1 and Th2 cell differentiation pathways	cNK cells: significant cytotoxicity trNKp cells: strong proliferative capacity trNK1 cells: active ribosomal activity	(88)
mice	uterus	NK1:NK1a,NK1b NK2:NK2a,NK2b NK 3/ILC1 cell clusters	NK1 cells: high levels of EOMES,PRF1 and GZMB, high expression of genes encoding integrins ITGA1(CD49a),ITGAD (CD11d),ITGAX (CD11c) and adapter protein XB130) NK2 cells: high expression of KLRC1 (NKG2A),KLRC2 (NKG2C),KLRC3 (NKG2E), XCL1 and XCL2;low expression of KIR NK3/ILC1 cells: high expression of CD160,KLRB1 (CD161),CCL3,CCL4 and CCL5	—	(89)
mice	uterus	cluster0-9	Cluster 1:high expression of marker genes ENTPD1,CYP26A1 and B4GALNT1, and high expression of KIR2D1, KIR2D3, KIR3D1 and LILRB1, contain more cytoplasmic granules such as GNLY,GZMA,GZMB and GZMK Cluster 3:high expression of marker genes ANXA1 and ITGB2;low expression of KIR Cluster 4: high expression of marker genes C1orf162, RASGEF1B, and ZNF683	cluster 1: mature dNK cells that maintain the balance between immune tolerance and immune effector functions during pregnancy cluster 3: exhibit a pronounced cytotoxic tendency cluster 4: represent the necessary differentiation process for dNK cells to transition towards immune tolerance	(90)
human	bone marrow	cluster1-8	Cluster 1:CD56dim GZMK- NK cluster2: chemokines (CCL3, CCL4, XCL1) are present, but also includes GZMK and CD160 cluster4: high expression of SELL, CD2, and IL7R cluster5: low expression of NKG7 and IL7R cluster6: upregulation of cell cycle-related genes cluster7: higher expression of SELL (CD62L) compared to cluster 1, with lower expression of GZMB, PRF1, CD16 and FGFBP2 cluster8: high expression of NKG7 and FCGR3A, but has lower expression of PRF1 and GZMB	cluster1:CD56dim GZMK- NK cells cluster2: ltNK, lymphoid trNK cells cluster4: CD56 bright NK cells cluster5: ILCs cluster6: proliferating NK cells cluster7: CD56dim GZMK+ NK cells cluster8: CD56- CD16+ NK cells	(45)
human	bone marrow	CD56dimGZMK- NK:cluster1-7	cluster1: high expression of S100A4 and S100A6, with upregulation of genes ACTB, ACTG1, CORO1A, PFN1 involved in cytoskeletal remodeling cluster2: upregulate genes include DUSP2, CXCR4, and BTG1 cluster3: absent of CCL5 cluster4: high expression of NKG2A cluster5: high expression of FGFBP2 and PRSS23 (serine protease 23) cluster6: high expression of S100A4 and S100A6; upregulation of NKG2C; upregulation of ribosomal protein-encoding genes IL32, GZMH, and GNLY; downregulation of KLRC1, KLRB1 (CD161), and CD160	cluster 1: terminally differentiated NK cells with high cytotoxicity cluster6: terminally differentiated NK cells with the presence of adaptive-like CMV-associated cells	(45)
human	bone marrow	7 bone marrow derived clusters: “CD56brightNK” “Transitional NK” “Active NK” “Adaptive NK” “MatureNK” “Terminal NK” “Inflamed NK”	“CD56bright NK”: high expression of IL-7R, CD62L(SELL), NKG2A, GZMK, XCL1 and XCL2; low expression of FCGR3A and CD160 “Transitional NK”: moderate expression of marker genes for CD56bright and CD56dim CD57+ NK cells “Active NK”: high expression of NR4A2, DUSP1, FOSB, FOS, JUN, and JUNB genes “Inflamed NK”: upregulation of a gene set positively regulating IFN-γ signaling, Toll-like receptor (TLR) signaling, and inflammatory responses “Adaptive NK”: high expression of NKG2C “Mature NK”: high expression of molecules important for cytotoxic functions; high expression of cytolytic molecules including perforin and granzymes (GZMA, GZMB, GZMH); high expression of β-actin, actin-related protein 2/3 complex subunit 2 (ARPC2), coronin 1A (CORO1A), and CFL1; high expression of negative regulatory factors related to cytotoxicity such as cysteine protease inhibitor F (CST7) and profilin 1 (PFN1); upregulation of cell adhesion and signaling molecules “Terminal NK”: high expression of the transcription factor ZEB2, increased expression of CX3CR1 and HAVCR2 (TIM-3), and depletion of ribosomal genes	“Inflamed NK”: positively regulate the inflammatory responseto interferon stimulation “Active NK”: rapidly induced in response to stimulation “Adaptive NK”: positively regulate a gene set involved in the transport of vesicles from the endoplasmic reticulum to the Golgi apparatus, as well as a gene set for IFN-γ production “Mature NK”: cytotoxic functions	(84)
human	peripheral blood	7 blood NK subgroups: cluster0.1.2.3.4.6.8	cluster0: high expression of chemokines CCL4, CCL3, CCL4L2, and CCL3L3 cluster1: high expression of TNFRSF9 (4-1BB) cluster2: higher expression of lytic granule gene FCGR3A (CD16) cluster3: lack expression of SIGLEC7, reduced expression of KLRC1 (NKG2A) and natural cytotoxicity receptors, releases large amounts of chemokines CCL3 and CCL4, and significantly more CCL5 cluster4: high expression of GPR183, IL7R, LTB, GZMK, CD62L, CCR7, CD2 and KLRC1 (NKG2A); low expression of FCGR3A (CD16), and very little or no PRDM1 cluster6: high expression of KLRC1 (NKG2A), IL2RA, XCL1, XCL2, FCGR3A (CD16), GZMA, PRF1, GNLY, and GZMB cluster8: significant loss of ribosomal expression, moderate enrichment of autophagy-related markers, and significantly lower numbers of expressed genes and unique molecular identifiers	cluster0, 1: CD56dim NK cells cluster2: terminally differentiated CD57+ NK cells cluster3: CD56-CD16+CD7+ NK cells cluster4: CD56bright NK cells cluster6: CIML NK cells cluster8: cells that have undergone senescence or death	(91)
human	peripheral blood	3 clusters	cluster1: CD56-CD16highHLADR+ mature NK cells cluster2: mature subpopulation of CD56dimCD16+ NK cells cluster3: immature subpopulation of CD56brightCD16− NK cells	cluster1: related to chronic HIV infection	(92)

### 3.2 Advances in the study of NK cell heterogeneity in the tumor microenvironment through scRNA-seq technology

**Figure 4:**
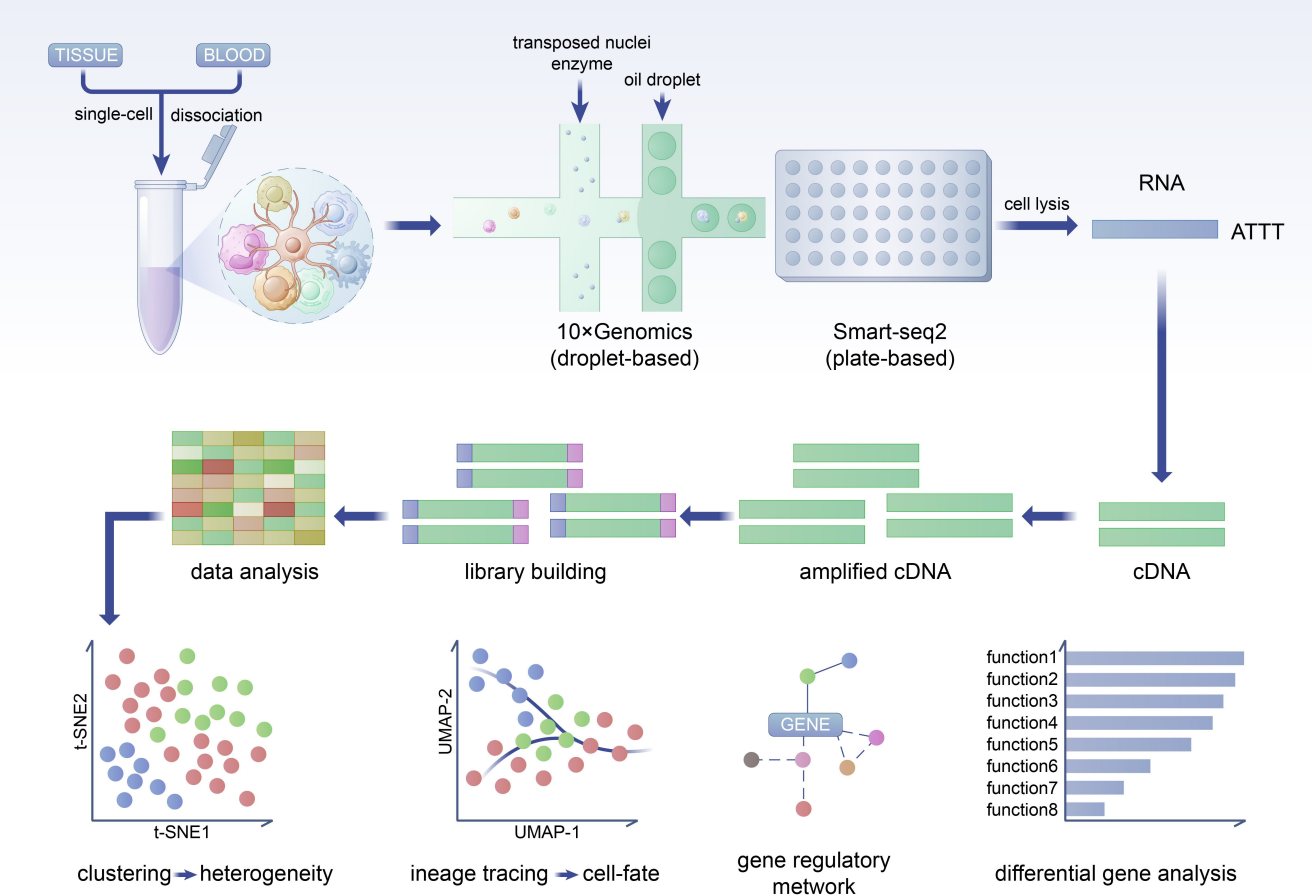
NK cells in tumor tissues exhibit expression differences not only compared to peripheral blood but also show significant differences compared to adjacent normal tissues. This disparity underscores the necessity to focus on the distinctions between tumor tissues and adjacent normal tissues. The diversity of subpopulations within tumors and the heterogeneity of expression among different tissues contribute to the overall heterogeneity of NK cells in the TME.

Traditional methods are insufficient for accurately assessing the functional state of individual NK cells within tumors. Therefore, higher resolution techniques are necessary to delineate the heterogeneity of NK cell subsets, their spatial distribution, and functional status within TME. scRNA-seq possesses the capability to identify specific cell populations and subpopulations, making it a potent tool for analyzing the heterogeneity present in the TME (42). (**Figure 4**).

#### 3.2.1 Functional analysis of NK cell subsets and identification of new subgroups

scRNA-seq enables the detection of heterogeneity between cells, allowing for a more detailed classification of NK cells that is not achievable with bulk sequencing methods. (**Table 3**) Recent studies have utilized scRNA-seq to analyze the pan-cancer landscape of NK cells. Tang F et al. identified five subgroups of CD56^bright^CD16^low^ NK cells and nine subgroups of CD56^dim^CD16^high^ NK cells and compared them with adjacent non-tumor tissues. The study revealed reduced expression of the cytokines CCL3 and CCL4 in the c3-CCL3 NK cells of tumor tissues and decreased expression of XCL1 and XCL2 in the c5-CREM NK cells, indicating their functional transition within tumors (100). Xu L et al. characterized the heterogeneity of NK cells in the breast TME under single-cell analysis, categorizing NK-0 to NK-5 as “memory-like” NK cells, NK cells with reduced IFN-γ production, CD56^dim^ bone marrow NK cells, tissue-resident NK cells, activated NK cells involved in direct anti-tumor responses, and NK cell activity inhibitors (101). Song G et al. sequenced NK cells in HBV/HCV-related hepatocellular carcinoma and non-tumor liver tissue, and divided them into six subgroups (NK_c1, 2, 3, 4, 5, 6). Based on differential gene expression, they defined an terminal NK subgroup (NK_c1), an exhausted NK subgroup (NK_c4), and two undefined NK subgroups (NK_c2 and NK_c6) and noted that NK_c5 was absent in the non-HBV population and played an additional antiviral role (44). Liu H et al. used single-cell sequencing to compare NK cells in liver cancer with NK cells in normal liver tissue and found that the L3-NK-HLA and L4-LrNK-FCGR3A subgroups were absent in tumor tissues, while L2 and L5 subgroups were significantly increased. They observed high expression of inhibitory receptor genes TIGIT, NKG2A, TIM3, and CD96 in L1-NK-CD56^bright^ cell, TIM3 and CD96 in L2-NK-CD56^dim^ cells, and KLRC1 and TIGIT in L5-LrNK-XCL1 cells. Furthermore, common tumor-related genes (RHOB, TALDO1, TKT, and HLA-DPA1) are overexpressed in L5-LrNK-XCL1 cells, exhibiting inhibitory anti-tumor activity (87). A study re-clustered all NK cells from 40 tumor samples and adjacent normal tissues in non-small cell lung cancer (NSCLC) patients into 12 subgroups (c0-c11), showing differential expression of cytotoxic markers and varying enrichment in lung squamous cell carcinoma(LUSC) and lung adenocarcinoma(LUAD) (88). Another study conducted cluster analysis on NK cells in triple-negative breast cancer (TNBC), HER2^+^ and luminal-like breast cancer and divided them into six groups. They defined two cytotoxic NK cell subgroups and two exhausted NK cell subgroups as well as two NKT cell groups. The study found that TNBC had a higher proportion of cytotoxic NK cells and an increased expression level of “cytotoxic” marker genes (GZMA, GZMB, and CST7) in NK_c03_Cytotoxic_GZMB while HER2^+^ and luminal-like breast cancer lost this feature, which provided new insights for NK cell immunotherapy (89). Additionally, de Andrade LF et al. sequenced NK cell population in melanoma metastases and defined seven NK cell subgroups. They then categorized them into proliferating NK cells, high cytotoxic NK cells and low cytotoxic NK cells based on their expression (90).

**Table 3:** The classification of NK cell subpopulations and their corresponding functions in different tumors under single-cell sequencing technology.

Type of tumor	Subpopulation of NK cells	Gene expression or receptor function	Function	Reference
Hepatocellular carcinoma	L1-NK-CD56bright L2-NK-CD56dim L5-LrNK-XCL1	L1- NK-CD56bright: high expression of inhibitory receptor genes NKG2A, TIGIT, TIM3, and CD96 L2- NK-CD56dim: high expression of inhibitory receptor genes TIM3 and CD96 L5- LrNK-XCL1: high expression of inhibitory receptor genes KLRC1 and TIGIT;overexpression of common tumor-associated genes RHOB, TALDO1, HLA-DPA1, and TKT	reduced anti-tumor activity	(94)
Hepatocellular carcinoma	NK_c1, 2, 3, 4, 5, 6	NK_c1, 4, 6: CD56dim NK cells, strongly express cytotoxicity-related genes such as GZMB, GNLY, FGFBP2, and CD16, with higher expression of granzyme B, KIR2DL1, and CX3CR1 NK_c1: marker genes include LAIR2, IGFBP7, and CD55 NK_c4: marker genes include LAG3, PTMS, and S100A6 NK_c6: marker genes include MYOM2, CX3CR1, and PRF1 NK_c2, 3, 5: CD56bright NK cells, express higher levels of GZMK, CXCR6, and CD69 NK_c2: marker genes include TOX2, CXCR6, and XCL1 NK_c3: exsist in non-tumor liver tissue with high expression of FOS, FOSB, FOXP1, and ATF4, as well as NFKBIA and NFKBIZ which are two genes involved in NF-κB pathway NK_c5: exsist in non-tumor liver tissue with high expression of FOS, FOSB, FOXP1, and ATF4, as well as NFKBIA and NFKBIZ which are two genes involved in NF-κB pathway and specifically expresses antiviral-related genes such as IFI44, IFI44L, and MX1, with high expression of STAT2, IRF9, and IRF7	NK_c1: terminal NK cell NK_c2: undefined NK_c3: non-tumor tissue CD56bright NK cells NK_c4: exhausted NK cells NK_c5: absent in non-HBV populations, additional antiviral activity NK_c6: undefined	(95)
Lung cancer	all NK cells of 40 tumor samples and matched adjacent normal tissues from NSCLC patients 12 subclusters: c0-c11	C0 cells: highly express CD16 and exhibit high expression of the cytotoxic marker TYROBP C2 cells: higher expression of pro-inflammatory factor CCL4L2 compared to C1 cells C4 cells: highly express CD16 and show increased expression of TRDC C5 cells: express resting NK cell markers such as AREG, XCL1, and KLRC1 C9 cells: express CX3CR1 and NKG7	C0 and C1 cells: enriched in LUAD tissue C2 and C4 cells: abundant in LUSC tissue C5 cells: enriched in tumor tissue C9 cells: enriched in normal tissue	(96)
breast cancer	NK subpopulations in TNBC and HER2+ and luminal breast cancer: NK_c01: naive NK(SELL) NK_c02: activated NK(CD69) NK_c03: cytotoxic NK(GZMB) NK_c04: exhausted NK(HAVCR2) NKT_c05: activated NK(CD69) NKT_c06: exhausted NK(LAG3)	NK_c03: elevated expression levels of cytotoxic marker genes GZMB, GZMA, and CST7 NK_c04: high expression of exhaustion marker gene HAVCR2 NKT_c05 and NKT_c06: high expression of T cell marker genes CD3G and NK cell marker genes GNLY and NKG7	NK_c03: vigorous biological activity NK_c04: an exhausted state NKT_c05 and NKT_c06: NKT cells	(97)
melanoma	tNK.0, tNK.1, tNK.2, tNK.3, tNK.5, tNK.6, and tNK.7 in melanoma metastatic lesions	tNK.6: expression of cell cycle genes, including PCNA and MKI67 tNK.0, tNK.3, tNK.6, and tNK.7: high expression of chemokine genes XCL1 and XCL2 tNK.1, tNK.2, tNK.5, and tNK.6: high expression of CCL3, CCL4, CCL4L2, and CCL5 tNK.2 and tNK.5: highest expression levels of genes encoding cytotoxic proteins GZMA, GZMB, GZMH, GZMK, GZMM, PRF1, GNLY, and NKG7	tNK.6: proliferating NK cells tNK.0, tNK.3, and tNK.7: lower cytotoxicity tNK.1, tNK.2, and tNK.5: higher cytotoxicity	(98)
breast cancer	NK-0,NK-1,NK-2,NK-3,NK-4,NK-5	NK-0: high levels of CD16 and cytolytic molecules, enriched in KLRC2, ETS1, and effector genes GZMH and CCL5 NK-1: high expression of JUN, FOS, DUSP1 and genes associated with the NR4A family NK-2: high levels of CD16 and cytolytic molecules, increased expression of cytotoxic-related genes GZMA, GZMB, PRF1, SPON2, and S1PR5 NK-3: upregulated expression of SELL, IL7R, and GZMK, decreased expression of cytolytic genes and CD16 NK-4: increased expression of genes involved in interferon signaling IFI6 and ISG15 NK-5: reduced expression of cytolytic genes and CD16, increased expression of KLRC1 and CD96	NK-0: memory-like NK cells NK-1: NK cells with reduced IFN-γ production specificity NK-2: CD56dim bone marrow NK cells NK-3: tissue-resident NK cells NK-4: activated NK cells involved in direct anti-tumor responses NK-5: inactivators of NK cell activity	(99)

Additionally, single-cell sequencing technology can also identify new subgroups, thereby laying the foundation for a deeper understanding of tumor microenvironment changes. As previously mentioned, Tang F et al. (100) identified a c6-DNAJB1 subgroup (TaNK cells) specifically enriched in tumors. This subgroup exhibited significantly higher expression of transcripts such as KLF6 and EGR3, which are associated with the inhibition of cytotoxic function and inferred to represent a terminal state based on RNA velocity analysis. A high abundance of TaNK cells in tumors is associated with poor prognosis in cancer patients. Ding S et al. (89) observed a unique subgroup of SOCS3 ^high^CD11b^-^CD27^-^ immature NK cells that were found only in TNBC samples. These cells exhibited reduced cytotoxic granule characteristics and were responsible for activating cancer cells through Wnt signaling pathway in mice, thereby promoting tumor progression. Another study revealed that peripheral circulating NK cells differentiate along two distinct pathways within the tumor microenvironment, leading to different final states. One of these pathways results in a phenotype similar to intraepithelial ILC1s, which represents the NK cell phenotype with the highest anti-tumor activity (91). Furthermore, Lin Q et al. (92) discovered a CD8^+^ NK cell population specific to the TME of nasopharyngeal carcinoma, providing new insights into the complexity of the immune landscape in nasopharyngeal carcinoma.

#### 3.2.2 Functional heterogeneity of NK cell populations within TME

Single-cell RNA sequencing has the capability to generate single-cell level maps of TME, thereby providing the most comprehensive composition and transcriptomic information of immune cell populations. By utilizing scRNA-seq, researchers can summarize and analyze the differences between tumor and normal tissue, the diversity of TME maps across different subtypes of the same tumor, and the variations within different regions of tumors and at various stages of tumor progression.

Xiao J et al. (102) utilized spatial and single-cell transcriptomics to uncover the tumor heterogeneity of colorectal cancer, revealing that the proportion of NK cells and B cells in tumors was reduced by approximately 0.3-0.4 times compared to normal tissue. A study on melanoma showed that cell clusters with shared key genes and similar transcriptional characteristics had significantly different proportions in tumors. Tumor-infiltrating NK cells exhibited significantly higher expression of FOS and JUN compared to blood NK cells, and their expression of the KLRC1 gene (encoding the inhibitory receptor NKG2A protein) also elevated (90). In lung cancer, the c5 cell cluster enriched in lung cancer tissues expressed resting NK cell markers XCL1, KLRC1 and AREG, while the c9 cell cluster expressing CX3CR1 and NKG7 was abundant in normal tissue (88). In lung adenocarcinoma lesions, NK cells were the most abundant immune cell lineage compared to non-affected lung tissue (nLung), with the CD16^+^ NK cell subset showing the most significant reduction. NK cells infiltrating tumor lesions expressed higher levels of CXCR3, which is necessary for NK cell infiltration, while lower levels of GZMB and CD57, and reduced IFN-γ production, indicated their decreased cytolytic activity (99). Furthermore, overall exhaustion of NK cells was observed in neuroblastoma and lung adenocarcinoma metastasis, indicating that the tumor immune response shifts towards immunosuppression (94, 95).

Additionally, related studies have also focused on the differences in TME maps between different subtypes of the same tumor category. Studies have demonstrated significant differences in the distribution and expression of NK cells in various types of lung cancer, including LUAD and LUSC. Specifically, c0 and c1 NK cell clusters were abundant in LUAD tissues, while c2 and c4 NK cell clusters were enriched in LUSC tissues (88). De Zuani M et al. also observed reduced cytotoxicity of NK cells in LUAD and LUSC, with significant differences in the co-expression of various immune checkpoint inhibitors (96). A study on human lung adenocarcinoma analyzed two distinct microenvironmental patterns formed by the transcriptomes of heterogeneous cancer cells. The inert N3MC microenvironment, compared to the immunologically activated CP2E microenvironment, contained normal-like myofibroblasts, non-inflammatory monocyte-derived macrophages, NK cells, bone marrow-derived dendritic cells, and conventional T cells, and was associated with a favorable prognosis (83). A study on mouse gliomas revealed increased infiltration of CD4^+^ T cells, CD8^+^ T cells and NK cells in low-grade gliomas (LGGs), while high-grade gliomas (HGGs) lacked this infiltration (98). Conversely, the abundance of neutrophils, T cells, and NK cells in samples from human high-grade gliomas showed an increasing trend compared to low-grade gliomas (103), suggesting that this trend may be related to the staging and malignancy of tumor progression and is independent of species differences. Ding S et al. (104) analyzed tumor samples from patients with TNBC and non-TNBC, finding that HER2^+^ and luminal-like breast cancer exhibited significantly lower levels of cytotoxic NK cells compared to TNBC, indicating a pro-tumor environment of NK cell exhaustion, and suggesting that these patients may benefit more from NK-based immunotherapy.

Furthermore, the enrichment and expression of NK cell subpopulations vary across different spatial areas within tumor tissue. A study examining the immune microenvironment of cervical squamous cell carcinoma (CSCC) revealed that different tumor areas exhibited varying degrees of cell infiltration. High-metabolic tumor areas displayed stronger signals for CD56^+^ NK cells and immature dendritic cells, while low-metabolic tumor areas showed stronger signals for eosinophils, immature B cells, and Treg cells (97). A single-cell study on nasopharyngeal carcinoma by Chen YP et al. (105) demonstrated that immune cells, particularly CD57^+^ NK cells, CD20^+^ B cells and IL3RA^+^ plasmacytoid DCs (pDCs), were more abundant in the tumor stroma compared to tumor epithelial nests.

What’s more, the level of NK cell infiltration and expression is dynamic and changes with tumor progression, recurrence, and metastasis. Studies have shown that chromosomal instability (CIN) in the precancerous stage can activate atypical NF-κB signaling, leading to anti-tumor immunity by NK cells. However, when CIN exceeds a certain threshold, the functional capacity of NK cells is compromised, and their effector capabilities are diminished compared to tumors with low CIN, thus creating a permissive microenvironment for tumor growth (104). Zhu J et al. characterized the cellular map of lung adenocarcinoma invasion trajectories through integrated analysis of single-cell RNA sequencing and spatial transcriptomics. The study revealed that cancer cells, NK cells, and mast cells were co-located in the cancer area in adenocarcinoma *in situ* (AIS), and NK cells and B cells increased during the progression from AIS to minimally invasive adenocarcinoma (MIA), enriching the study of cell ecology and spatial niches involved in the dynamic and sequential processes from AIS to MIA and subsequent invasive adenocarcinoma (IAC) (106). Tumor recurrence and metastasis are associated with shifts in the TME. In the study of chordomas, it was observed that NKT and NK cells had higher scores in recurrent chordomas compared to primary chordomas, and NK cells exhibited higher exhaustion scores in primary chordomas, providing a more comprehensive understanding of the microenvironment of primary and recurrent chordomas (107). Deng JY et al. analyzed the transcriptomic characteristics of metastatic NSCLC and found that the lung lesion group was enriched in NK cells and NK-mediated cytotoxicity and T-NK cell receptor signaling pathways, with NK cells, helper T cells, IFN-γ-related T cells, and proliferating T cells exhibiting higher cytotoxic activity than that of liver metastasis group (108). Similarly, the number and proportion of immune cells with tumor immune function, such as CD8^+^ T cells, NK cells, and monocytes, were significantly reduced in prostate cancer lymph node metastases compared to primary prostate cancer lesions (109). Gene mutation subtypes also influence cellular infiltration and transcriptional characteristics. BRAF mutant melanomas showed a significant reduction in the infiltration of CD8^+^ T cells and macrophages compared to BRAF wild-type melanomas, but an increase in the infiltration of NK cells, B cells, and NKT cells (110).

#### 3.2.3 NK cell-related intercellular interaction

The dynamic and heterogeneous nature of the tumor microenvironment contributes to insufficient infiltration and diminished efficacy of NK cell immunotherapy, which is a significant factor in tumor progression. Understanding the interactions between NK cells and other cells in the TME provides new insights for optimizing NK cell immunotherapy. Single-cell sequencing technology presents complex interactions of NK cells in a higher dimension, providing crucial insights for the selection of NK cell immune checkpoints and the development of drug intervention strategies.

**Figure 5:**
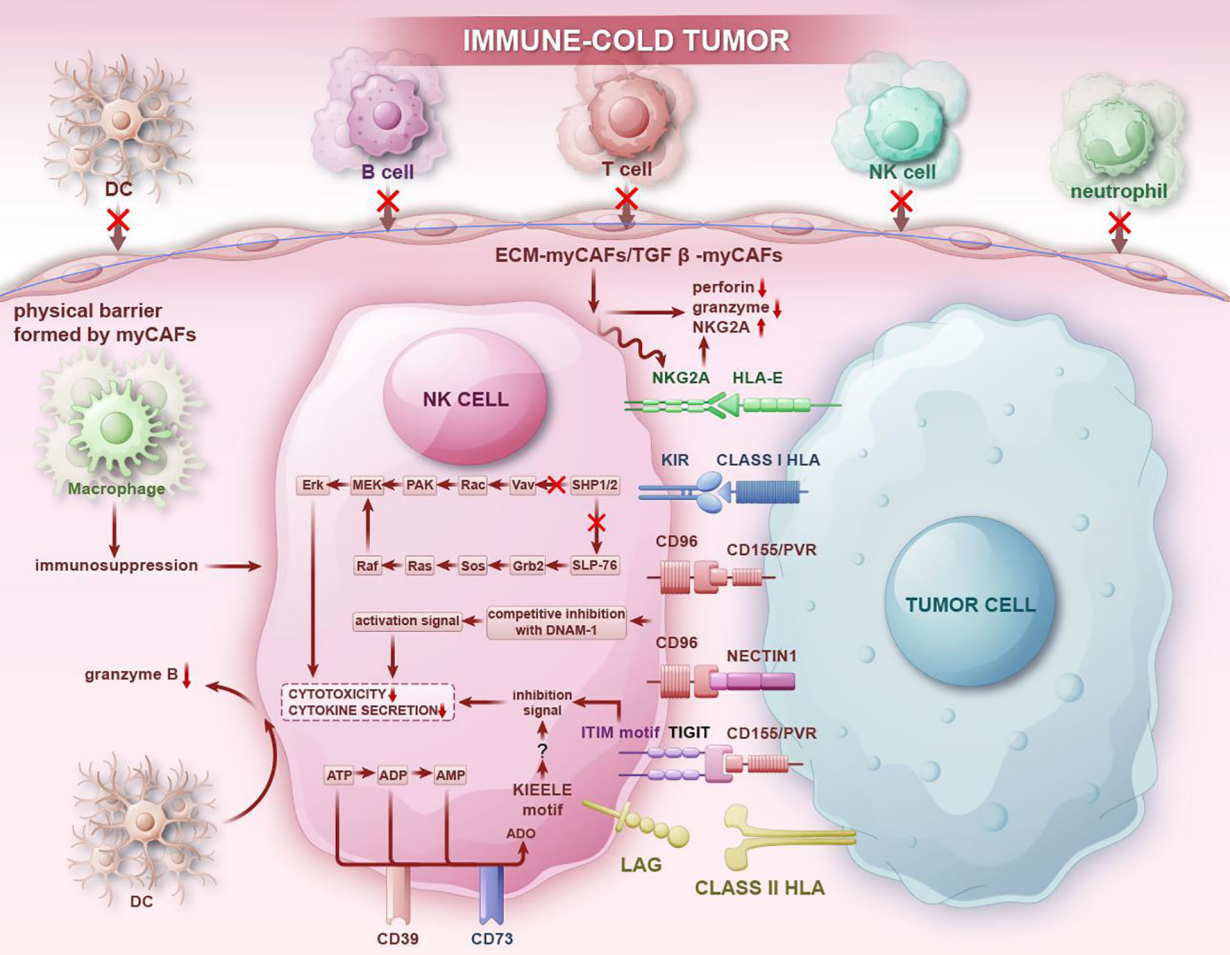
In immune-cold tumor regions, NK cells transmit immunosuppressive signals through immunosuppressive receptors and interact with immune cells in the immunosuppressive TME, thereby reducing the secretion of cytokines and decreasing cytotoxic activity, allowing tumors to evade NK cell attacks. At the same time, interactions between NK cells and other immune cells inhibit the chemotaxis of other immune cells, promoting tumor progression and the formation of an immunosuppressive microenvironment.

Firstly, interactions between NK cells and tumor cells can inhibit the cytotoxic function of NK cells, enabling tumor cells to evade immune surveillance and promoting tumor recurrence and metastasis. (**Figure 5**) NKG2A, an inhibitory receptor on NK cells, acts as an immune checkpoint receptor. Jiang H et al. (111) utilized single-cell sequencing technology to reveal that NK cells exhibited the highest expression of NKG2A, with over half of NK cells in TME expressing KLRC1. Malignant cells interact with NK cells through the HLA-E-NKG2A pathway, which presents new opportunities for the clinical treatment of gastric cancer. In the study of pancreatic ductal adenocarcinoma circulating tumor cells (CTCs), Liu X et al. also demonstrated that CTCs interact with NK cells via the immune checkpoint molecule HLA-E-NKG2A, protecting CTCs from NK-mediated immune surveillance and thereby promoting the metastasis of pancreatic ductal adenocarcinoma (112). CD39, a transmembrane protein encoded by the ENTPD1 gene, in synergy with CD73, can convert ATP and ADP into the immunosuppressive adenosine through a cascade reaction. Liu L et al. (113) found that the expression of CD39 and LAG3 of NK cells was significantly upregulated in most tumor samples, creating an immunosuppressive state in the TME. Cao G et al. (114) conducted cell interaction analysis to investigate the potential mechanisms underlying T/NK cell exhaustion and identified poliovirus receptor (PVR)-like protein signaling as the primary co-inhibitory interaction between NK cells (CD96 and TIGIT) and malignant cells (PVR/CD155 and NECTIN1). Recent single-cell RNA sequencing data suggests that high expression of platelet-related genes in circulating tumor cells CTC directly upregulates CD155 expression on tumor cells through platelet adhesion. This upregulation protects CTCs from NK cell killing through the interaction between CD155 and the immune receptor TIGIT, thereby promoting the metastatic dissemination of tumor cells (115). The functional state of NK cells in the TME is significantly suppressed, while unsuppressed NK cells can exert cytotoxic effects and resist tumors. A study using scRNA-seq to analyze various immune cells within tumors found that NK cells in tumor sites expressed cytotoxic molecules such as GZMA, PRF1, and chemokines XCL2, CCL5, CCL3, and CCL4, indicating the potential for an antitumor response. Additionally, NK cells also expressed several inhibitory and co-stimulatory molecules, including CD96, TNFRSF18, and KIR2DL4, representing targets for regulating their function (116).

NK cells can also interact with immune cells, forming a complex immune network that together constructs an immunosuppressive tumor microenvironment. The study of NK cell-immune cell interactions provides foundational information for deepening the understanding of the immune network and immunotherapy. Research has found a strong negative correlation between the frequency of STAB1^+^ Mφ/AI Mφ and T/NK cells, further validating that Mφ acquire an immunosuppressive phenotype and inhibit NK cell infiltration in non-small cell lung cancer (96). Using single-cell transcriptomic analysis, Kim HJ et al. (117) found that CD169^+^ macrophages in human and mouse gliomas produce pro-inflammatory chemokines which can lead to the accumulation of NK cells, and NK cell-derived IFN-γ is crucial for the accumulation of CD169^+^ macrophages derived from blood monocytes in gliomas. In a single-cell sequencing study of primary tumors and adjacent non-tumor samples from different organs or tissues and metastases, Jiang H et al. (111) found that when macrophages and NK cells were co-cultured, treatment with an anti-NKG2A antibody inhibited HLA-KLRC1 (NKG2A) signaling and the expression of IFN-γ was significantly improved. Additionally, researchers showed through scRNA-seq of glioblastoma tumor-infiltrating immune cells that the loss of Dicer in macrophages reduced the proportion of cluster 3 of glioma-associated macrophage (GAM) and reprogrammed the remaining GAM to a pro-inflammatory activation state, interacting positively with tumor-infiltrating T cells and NK cells through TNF paracrine signaling, creating a pro-inflammatory immune microenvironment for tumor cell elimination (112). A study compared receptor-ligand pairs in LUAD and LUSC to evaluate the interaction strength between immune cell subsets in the tumor microenvironment and found that the SPP1 pathway showed greater interaction between SPP1-Mφ clusters and NK cells in LUSC than in LUAD, and further analysis revealed different pathways in LUSC, such as TIGIT and cell proliferation-related LCK, which can enhance the interaction between NK cells and SELENOP-Mφ together with SPP1-Mφ clusters (88). Similarly, the interactions between NK cells and other immune cells, including cancer-associated fibroblasts (CAF) and dendritic cells, have been thoroughly investigated. A study deconvoluted spatial transcriptomics at the single-cell level and performed functional analysis to generate a high-resolution integrated map of breast cancer. The study found that co-culture of FAP^+^ CAF clusters with NK cells reduced the levels of perforin and GZMB in NK cells and significantly increased the proportion of CD56^high^ NK cells and their surface NKG2A levels, thereby reducing the cytotoxicity of NK cells (118). Additionally, Ou Z et al. used single-nucleus RNA sequencing (snRNA-seq) and spatial enhanced resolution omics sequencing to study the immune microenvironment of CSCC and found that the number of B cells, T cells, NK cells, neutrophils, DCs and Th1 cells was significantly reduced in myCAF^+^ tumors, proving that myCAF may act as a physical barrier to prevent the infiltration of pro-immune cells (97). What’s more, mature cDCs (LAMP3^+^ DCs) which are recently characterized showed the strongest interaction with CD56^dim^ NK cells. Tumor-infiltrating LAMP3^+^ DCs showed lower IL-15 expression compared to those in non-tumor tissues, and NK cells physically close to LAMP3^+^ DCs expressed lower levels of GZMB, suggesting that LAMP3^+^ DCs have a compromised activation effect on CD56^dim^ NK cells in TME (100).

## 4 Drug intervention and resistance mechanism research guide new treatment strategies

### 4.1 Pharmacological and non-pharmacological intervention mechanisms

Recent studies on drug mechanisms have shown that certain pharmacological and non-pharmacological interventions can inhibit tumor growth, reduce tumor invasion, and improve prognosis. This effect is closely associated with the activation of NK cells within the tumor microenvironment and the interactions between NK cells and other cells. Notably, combination therapy also demonstrates significant advantages.(**Table 4**).

**Table 4:** The application of single therapy and combination therapies influences activation of NK cells within the TME, thereby affecting the therapeutic efficacy against tumors.

Type of tumor	Drug/dose	Drug combination	Source (Human/animal)	Sample/subject	Outcome	Reference
esophageal squamous cell carcinoma(ESCC)	4 Neoadjuvant ICB	chemoradiotherapy	human	6 adjacent normal esophageal mucosa and 26 tumor samples	Immune checkpoint blockade enhanced CD16+ NK cell proportion and augmented NK cell activation and cytotoxicity	(119)
melanoma	microbiota-derived stimulator of interferon genes (STING) agonists	ICB therapy	human/mice	several preclinical cancer models	microbiota-derived stimulator of interfero ngenes (STING) agonists induce IFN-I production by intratumoral monocytes to regulate macrophage polarization and natural killer (NK) cell-DC crosstalk	(113)
melanoma	anti-PD1 treatment(nivolumab)	anti-LAG3 treatment(relatlimab)	human	blood samples from 40 immunotherapy-naïveor prior immunotherapy-refractory patients with metastatic melanoma treated with anti-LAG3+anti-PD1 in a phase I trial (NCT01968109)	stimulate NK cells, and especially in adaptive NK cells, an increase in IFN-γ response, upregulation of cytotoxicity associated genes, and elevated degranulation and cytokine production were observed	(120)
non-small cell lung cancer	neoadjuvant PD-1 blockade	chemotherapy	human	transcriptomes of 92,000 single cells from 3 pre-treatment and 12 post-treatment patients with non-small cell lung cancer (NSCLC)	expansion and activation of cytotoxic T cells and CD16+ NK cells, reduction of immunosuppressive Tregs, and activation of memory CD8+T cells into an effector phenotype	(121)
pancreatic tumor	Xkr8 siRNA	a prodrug conjugate of 5-fluorouracil and oxoplatin (FuOXP)	human	orthotopic pancreatic tumor model	increased infiltration of proliferative NK cells and activated macrophages in TME	(122)
hepatocellular carcinoma	2,5-dimethylcelecoxib (DMC)	—	human	HCC mice treated with DMC, celecoxib and MK-_x005f886 (a known mPGES-1 inhibitor)	enhance antitumor activity of natural killer (NK) and T cells;inhibit exhaustion of NK and T cells	(123)
melanoma tumour	r docosahexaenoic acid(DHA)	—	mouse	mouse B16F10 melanoma tumour model	enhance NK-cell IFN-γ production and cytotoxicity	(124)
pancreatic cancer liver metastasis (PCLM)	α-galactosylceramide (αGC)	—	mouse	over 30,000 immune cells from normal liver and PCLM with or without αGC treatment	increased cytotoxic activity of iNKT/NK cells, increased transcription of Gzma, Gzmb, and Prf1(encodes Perforin)	(125)
melanoma, lung carcinoma, colon cancer	three different nitric oxide-releasing compounds (SNAP, SNP, and ISMN)	—	mouse	tumor-bearing mouse models	increase the population of CD8+ cytotoxic T cells and NK cells	(126)
subcutaneous tumor, orthotopic bladder cancer	inhibition of CD39 (CD39i)	—	mouse	subcutaneous tumor model and orthotopic bladder cancer model	increase the proportion of tumor infiltrated NK cells, cDC1 and cycling CD8 + T cells	(127)
colon cancer	HSV-2-based oncolytic virus OH2	—	mouse	peripheral blood from tumor-bearing mouse models	the cytotoxic ability of peripheral cytotoxic CD8+ T cells and mature NK cells was elevated	(128)
pulmonary metastatic melanoma	noninvasive radiofrequency radiation (RFR)	—	mouse	model of pulmonary metastatic melanoma (PMM)	upregulated the expression of genes related to NK cell activation, cytotoxicity, as well as lymphocyte chemotaxis, such as Klrk1, Gzma, and Ccl5	(129)
early-stage breast cancer	microwave ablation (MWA)	—	human	peripheral blood mononuclear cells (PBMCs) from six patients	systemic NK and CD8+ T cells were activated with enhanced the cytotoxic activity and chemokine activity	(130)

#### 4.1.1 Immune checkpoint inhibitors

Immune checkpoint blockade (ICB) can disrupt the immune surveillance of cancer cells and has significantly transformed the landscape of cancer treatment (131). A research conducted single-cell RNA and T cell receptor sequencing on PBMCs from 60 patients with stage IV non-small cell lung cancer and divided NK cells into CD56^high^ NK cells (c1), CD56^low^ NK cells (c2), and CD56^low^ NK_IFNG cells (c3). The results revealed that after immune checkpoint inhibitor (ICI) treatment, the proportion of the c1 subgroup increased, and in the durable clinical benefit (DCB) group after ICI treatment, the proportion of the c3 subgroup also increased (132). Another study on esophageal squamous cell carcinoma analyzed the dynamic changes in the tumor immune microenvironment and found that immune checkpoint blockade enhanced the activation and cytotoxicity of NK cells by increasing the frequency of CD56^dim^CD16^+^ NK cells (133). Shang J et al. (134)analyzed single-cell transcriptomic data from patients with non-small cell lung cancer treated with anti-PD-1and they observed six different NK cell clusters, among which immature NK cells were enriched in the responder group and expressed a series of marker genes associated with anti-PD-1 response and were related to antigen processing, Th1 activation, Th17 cell activation and other immune regulatory processes. Additionally, scRNA sequencing also demonstrated that microbial-derived interferon gene stimulators (STING) agonists can regulate macrophage polarization, induce IFN-I production from intratumoral monocytes, and facilitate crosstalk between NK cells and DCs. The microbiome triggers the IFN-I-NK cell-DC axis within the tumor, improving the efficacy of ICB (135).

The combination of ICB therapy with other treatment modalities has yielded promising results. Relatlimab combined with nivolumab (anti-LAG-3 and anti-PD-1) has been approved by the FDA as a first-line treatment for stage III/IV melanoma. Utilizing single-cell RNA and TCR sequencing in conjunction with other multi-omics analyses, researchers found that adaptive NK cells were enriched in responders after treatment and underwent significant transcriptomic changes, leading to an activated phenotype (136). Single-cell sequencing compared the transcriptomes of approximately 92,000 single cells from three pre-treatment and twelve post-treatment NSCLC patients who received neoadjuvant PD-1 blockade combined with chemotherapy and researchers concluded that the neoadjuvant PD-1 blockade combined with chemotherapy therapy can promote the expansion and activation of cytotoxic T cells and CD16^+^ NK cells (137). Additionally, a study on a new type of high-grade serous ovarian cancer (HGSC) compared samples treated with a bispecific anti-PD-1/PD-L1 antibody to a control group treated with monospecific anti-PD-1 or anti-PD-L1. The results demonstrated that the bispecific antibody induced superior cellular state changes in T cells and NK cells, uniquely prompting NK cells to transition from a quiescent phenotype to an active and cytotoxic phenotype. This transition enhanced the efficacy of immunotherapy for ovarian cancer (138). Another study identified 207 liver cancer NK cell marker genes based on single-cell sequencing, which are primarily involved in immune functions and found that the degree of immune cell infiltration and function in the low-risk group were higher than those in the high-risk group, indicating that the low-risk group is more suitable for ICI and anti-PD-1 treatment (139).

#### 4.1.2 Other biochemical methods

Some single-cell sequencing studies have shown that NK cells in the TME after chemotherapy show increased infiltration and enhanced killing efficacy, indicating good anti-tumor effects. A study conducted whole exome sequencing, large-scale RNA sequencing, and single-cell transcriptomics on paired pretreatment and treatment samples from gastric cancer patients and explained the remodeling of the TME after platinum chemotherapy, characterized by the recruitment of NK cells, the reduction of tumor-associated macrophages, the repolarization of M1 macrophages, and the increase in effector T cell infiltration (140). The scramblase Xkr8 plays a key role in promoting tumor immunosuppression and the use of Xkr8 small interfering RNA (siRNA) targeting Xkr8 combined with the chemotherapeutic prodrug FuOXP in the TME of *in situ* pancreatic tumors caused an increase in the infiltration of proliferative NK cells and activated macrophages which could enhance the antitumor immune response and showing good antitumor effects (141). Additionally, a study based on high-dimensional cell counting deeply analyzed the effects and mechanisms of 2,5-dimethyl-celecoxib (DMC) on the infiltration of immune cells in liver cancer. The study found that after using DMC, NK cells and T cells in the TME showed strong anti-tumor activity and effectively inhibited the exhaustion of NK cells and T cells, thereby inhibiting the growth of liver cancer cells and improving the prognosis of mice (142).

The application of certain chemical agents has also been observed to affect NK cells through single-cell sequencing technology, providing insights into clinical efficacy and guiding treatment strategies. Wu S et al. concluded from single-cell sequencing in a mouse B16F10 melanoma model that docosahexaenoic acid (DHA) can enhance the effector function and oxidative phosphorylation of NK cells, effectively inhibiting the growth of B16F10 melanoma in the lungs (143). Yi Q et al. analyzed pancreatic cancer liver metastasis (PCLM) models with or without α-galactosylceramide (αGC) treatment, characterizing the overall changes in immune cells in the TME after αGC treatment. They found that the cytotoxic activity of invariant natural killer T cells (iNKT) and NK cells increased (119). Li CY et al. studied the immune mechanisms of the anti-tumor effects of low-dose nitric oxide donors in three ways, among which scRNA sequencing showed that low-dose nitric oxide donors increased the number of CD8^+^ T cells and NK cells while reducing central memory T cells (144). Through single-cell RNA sequencing, Liu L et al. found that sodium polyoxotungstate inhibited the increase of NK cells within tumors. They also performed basic analysis of NK cell phenotypes through flow cytometry, revealing that treatment with CD39i significantly increased the proportion of mature CD27^+^CD11b^+^ and CD27^-^CD11b^+^ NK cells, confirming that CD39 is a potential target for immunotherapy in bladder cancer (113).

Additionally, some treatment strategies using oncolytic viruses have led the way in biological treatment concepts. For example, in a mouse model treated with the oncolytic virus OH2, Wu Q et al. (120) found that OH2 could enhance the cytotoxic capabilities of peripheral CD8^+^ T cells and mature NK cells, effectively activate the systemic immune response and induce a sustained anti-tumor immune response.

#### 4.1.3 Non-pharmacological intervention mechanisms

Non-pharmacological interventions, such as radiation and microwave ablation therapy, have also been better explored for their efficacy through single-cell RNA sequencing. Jiao JZ et al. investigated the impact of non-invasive radiofrequency radiation (RFR) exposure on the tumor immunosuppressive microenvironment. ScRNA-seq analysis of infiltrating immune cells in lung metastatic melanoma revealed that RFR exposure could increase NK cell subsets, enhance the effector functions of tumor-infiltrating CD8^+^ T cells and NK cells, induce anti-tumor remodeling of TME and thereby inhibit tumor progression (121). Another study conducted scRNA-seq on PBMCs before and after microwave ablation (MWA) for breast cancer. The study found that XCL2^+^ NK cells specifically enriched after MWA were associated with genes related to cytokine production pathways, lymphocyte differentiation pathways and T cell activation pathways and presented higher chemokine function. While in GZMB^+^ NK cells, IFITM1, MYOM2, JUN, IFITM3, CCL4, and RBM25 were upregulated, indicating higher cytotoxic activity (145).

### 4.2 NK cell exhaustion as an important factor in the mechanism of tumor resistance

Immunotherapy and emerging treatments have revolutionized treatment for cancer but most patients either do not respond to immunotherapy or show resistance, and the underlying mechanisms remain to be fully elucidated. Single-cell sequencing technology has provided a robust technical foundation for us to gain a deeper understanding of the resistance mechanisms within the tumor microenvironment under various therapeutic interventions.

Zhang Y et al. discovered that patients with MET amplification exhibit resistance to ICB in lung cancer. Through deep single-cell RNA sequencing, they found that MET amplified patients exhibited immunosuppressive characteristics, characterized by the increase in XIST^+^ and CD96^+^ exhausted NK cell subsets and reduction in CD8^+^ T cells and NK cell populations. The study also demonstrated that inhibition of MET can overcome the decreased efficacy of ICB caused by MET amplification (146). A study identified a new podoplanin-positive (PDPN^+^) CAFs subset enriched in trastuzumab-resistant tumor tissues through single-cell sequencing. This subset induced trastuzumab resistance in HER2^+^ breast cancer by inhibiting the NK cell-mediated antibody-dependent cellular cytotoxicity (ADCC) immune response (147). Li XY et al. conducted scRNA sequencing on tumors of patients with metastatic melanoma or head and neck squamous cell carcinoma and found that NKG7 was highly correlated with the cytotoxicity of CD8^+^ T cells and NK cells. Tumors expressing new antigens grew faster in mice lacking NKG7. Furthermore, the efficacy of single or combined ICB was significantly reduced in NKG7-deficient mice (122). As previously mentioned, scRNA sequencing revealed that conditional deletion of HIF-1α in mouse tumor-infiltrating NK cells resulted in reduced tumor growth, increased expression of activation markers and effector molecules, and enriched the NF-κB pathway in tumor-infiltrating NK cells. Furthermore, long-term culture with a HIF-1α inhibitor also increased the efficacy of human NK cells (123). Additionally, through single-cell transcriptomics and gene reporter mice, Bi J et al. found that the expression of TIPE2 in the human and mouse TME was associated with NK cell exhaustion, and TIPE2 deletion promoted the anti-tumor activity of human NK cells in mice, enhancing NK cell infiltration and effector function, thereby improving the survival rate of tumor patients (124). A study using high-dimensional flow cytometry, mass cytometry, and single-cell RNA sequencing combined with functional analysis created a comprehensive map of human NK cell migration phenotypes and explained the reasons for the systemic rejection of NK cells from the tumor microenvironment, providing a basis for designing the next generation of NK cell therapy for malignant tumors (125). Drug resistance largely depends on the sensitivity and efficacy of different tumor subgroups to treatment. Through the integration of scRNA-seq data and TCGA data in the study of head and neck squamous cell carcinoma (HNSCC), Yang F et al. (126) found that, compared to the HPV^+^ HNSCC cohort, the IL6/IL6R and CCL2/CCR2 signaling pathways had a more significant impact on NK cells evading immune attack in the HPV- HNSCC cohort. The combination of CCR2 chemokine receptor antagonists with IL-6 blockers had a significant anti-tumor effect in HPV- HNSCC, which was associated with larger number of activated intratumoral NK cells.

### 4.3 Selection of therapeutic target

Through single-cell sequencing technology, researchers can also achieve the identification of cell markers, thereby providing better targets and ideas for drug treatment. In a pan-cancer analysis utilizing single-cell sequencing technology, Tang F et al. (127) identified regulator of G protein signaling 1 (RGS1) as a marker expressed specifically in NK cells within tumor and adjacent non-tumor tissues, while being nearly undetectable in blood. RGS1 was widely expressed across analyzed patients and cancer types, exhibiting higher sensitivity and specificity than other tumor tissue resident markers. Another study in mice identified CCRL2 as a marker of general alveolar and pulmonary capillary endothelial cells through scRNA-seq and found that upregulating CCRL2 increased NK cell recruitment and reduced lung tumor growth, thus establishing CCRL2 as an NK cell lung homing molecule and providing new insights into NK cell-mediated lung tumor immune surveillance (128). Zhang Y et al. investigated the potential role of pyroptosis-related genes (PRGs) in preventing malignant melanoma through the study of melanoma pyroptosis, identifying CD57^+^ NK cells with downregulated cell pyroptosis expression, particularly GSDMB^+^, as a protective prognostic predictor in clinical settings (129). A separate study demonstrated that the endoplasmic reticulum-regulated secretory protein SCUBE2 was enriched in osteoblasts within early bone metastatic niches, promoting osteoblast differentiation. Osteoblast deposition of collagen inhibited NK cells by inhibiting LAIR1 signaling, thereby promoting tumor colonization (130). Slattery K et al. (148) conducted single-cell analysis, metabolic flux, and confocal analysis on NK cells from patients with metastatic breast cancer and the healthy control groups, identifying TGF-β as a factor involved in the metabolic dysfunction of circulating NK cells in patients with metastatic breast cancer. Blocking the GARP-TGF-β axis can restore the metabolism and function of NK cells, representing an important target for enhancing NK cell immunotherapy.

## 5 Concluding remarks

Despite the continuous emergence of new technologies and a deeper understanding of interactions within tumors, information regarding the sources of NK cell heterogeneity, the individual developmental processes of NK cells, and the role of tumor programming in regulating the functional diversity of NK cells remains insufficiently comprehensive. With ongoing advancements in technology and the continual exploration of interactions within tumors, many questions may be addressed in the coming years, potentially providing promising targets for the development of candidate therapies. The application of single-cell sequencing technology offers a promising avenue to tackle these challenges. Single-cell sequencing technology plays a significant role in identifying new NK cell subpopulations, analyzing differences among various subpopulations, and investigating intercellular interaction relationships in depth. Single-cell RNA sequencing studies on NK cell heterogeneity have identified correlation maps between NK cell subtypes in the TME and tumor progression, providing evidence for patient prognosis and therapeutic efficacy. These studies reveal promising cellular and molecular markers as new targets for cancer treatment. They offer immune cell marker genes for tumor prognosis, which can serve as a reference for immunotherapy decisions in cancer patients. Additionally, scRNA-seq provides valuable insights into the transcriptional heterogeneity of the TME, the overall intercellular interaction network, and new molecular mechanisms of action and resistance related to immune checkpoint inhibitors and targeted therapies. Single-cell sequencing enables dimensionality reduction and clustering of NK cells into distinct subpopulations, each exhibiting unique transcriptional profiles. This facilitates a paradigm shift in prognostic stratification from population-level analysis to single-cell precision. Through scRNA-seq, we identify NK-specific immune checkpoints (e.g., NKG2A, KIR), which inform monoclonal antibody targeting strategies (e.g. anti-NKG2A monalizumab) for precision patient selection. Spatial proximity analysis of interactions between NK cells and tumor cells coupled with expression quantitation of receptor-ligand pair directly correlates with immune evasion capacity and metastatic potential, providing actionable biomarkers for personalized immunotherapies (e.g. CAR-NK design, checkpoint inhibitor combination). Furthermore, single-cell sequencing can also compare the intervention effects of regulatory molecule dynamics and metabolic reprogramming interventions, evaluate the superiority of synergistic immunotherapy to guide intervention measures, overcome tumor drug resistance and enhance antitumor efficacy.

At the same time, we must also acknowledge the limitations of scRNA-seq technology. Firstly, there are technical issues. RNA instability compromises library construction quality, and some key but rare subpopulations, such as drug-resistant cells and migratory cells, may escaped detection by most methods, including scRNA-seq. Secondly, conventional scRNA-seq captures cellular states but fails to preserve spatial information. The continuous improvements in spatially resolved techniques and multimodal data integration are expected to significantly advance our understanding of intercellular interactions and spatial effects in the coming years. Moreover, there are also challenges in clinical translation. Issues such as insufficient drug target potency, the need for multi-target combination to enhance anti-tumor ability, and limitations in adoptive cell therapies including poor *in vivo* persistence and insufficient tumor infiltration, all collectively impede translational progress in the field. David’s research summarized the eleven major challenges faced by single-cell data science, highlighting various issues with current single-cell sequencing data, such as insufficient sequencing coverage and depth, high overall costs, and sequencing biases arising from model construction and metric selection (149). We believe that the research benefits provided by single-cell sequencing technology will accelerate the standardization of analytical methods, thereby promoting technological development, while the accuracy and stability of sequencing data will continue to be optimized to meet the needs of clinical accessibility. The combination of single-cell sequencing technology with spatial transcriptomics can generate high-resolution tissue maps, delineate developmental pathways within specific spatial contexts, and elucidate gene expression activity in particular tissue regions, thereby enriching the dimension of single-cell sequencing technology (150).In recent years, NK cell-based cellular therapies, such as chimeric antigen receptor (CAR)-NK cell therapy, have demonstrated certain advantages over CAR-T cell therapy and have been partially validated for their roles in the treatment of various hematological malignancies (151).Single-cell RNA sequencing has been employed to evaluate the efficacy of CAR-NK cells in acute myeloid leukemia (AML). Studies revealed upregulation of cell proliferation, protein folding, immune response, and key metabolic pathways in CAR-NK cells, leading to tumor-specific, CAR-dependent activation and functionality against AML target cells (152). Furthermore, scRNA-seq has provided critical theoretical insights for gene-editing strategies in CAR-NK cells to counteract tumor immunosuppression in AML (153). Additionally, this technology enables comparative analysis of how agonists or cytokines influence CAR-NK cell activity and therapeutic outcomes. For instance, STING agonists were found to enhance the migration and cytotoxic capacity of mesothelin-targeted CAR-NK cells (154) and IL-15 helps overcome the loss of NK cell metabolic fitness induced by tumor interactions (155). Notably, ongoing innovations in single-cell sequencing methodologies and precision have not only strengthened the technical foundation for CAR-NK cell therapy applications but also provided unique insights into the multifaceted mechanisms underlying CAR-NK cell cytotoxicity (156, 157). Although research on CAR-NK cells in solid tumors remains limited, and their application faces multiple challenges, such as the degree of targeted infiltration, understanding NK cell biology is essential for the development of improved therapies, whether through the use of potential CAR-NK cells or by directly targeting NK cells. Therefore, we summarize the progress in the study of NK cell heterogeneity using single-cell sequencing to gain deeper insights into the biological characteristics of NK cells within the tumor microenvironment. Additionally, as recent studies frequently compare NK cell expression levels in the tumor microenvironment with those in peripheral blood to analyze the differences, we examine NK cell expression from both physiological and tumor-centric pathological perspectives. We also acknowledge the importance of studying NK cell expression in non-tumor tissues under physiological conditions. We also dissect the origins and developmental processes of tissue-resident NK cells and gather evidence regarding the enrichment of NK cell precursors in extramedullary tissues, providing new ideas and directions for NK cell-targeted therapies. We believe that the integration of various omics technologies, along with the construction of the latest data from cellular dynamics research, molecular profiling, and spatial localization, can create a platform for the development of new and more effective anti-tumor NK cell-targeted therapies. Excitingly, certain preclinical results are impressive and are expected to start generating clinical impact.

## Glossary

NK cells: Natural killer cells
TME: tumor microenvironment
scRNA-seq: single-cell RNA sequencing
HCL: Human Cell Landscape
ILCs: innate lymphoid cells
FcγRIII: Fcγ receptor III
KIR: killer cell immunoglobulin-like receptor
HSCs: hematopoietic stem cell
CLPs: common lymphoid progenitors
pre-NKPs: pre-NK progenitors
NKPs: NK progenitor cells
iNK: immature NK
LTis: lymphoid tissue inducers
CILPs: common innate lymphoid progenitors
CHILPs: common helper innate lymphoid progenitors
LTiPs: lymphoid tissue inducer progenitors
ILCPs: innate lymphoid cell precursors
IFN-γ: interferon-γ
AIR: adaptive immune receptor
trNK: tissue-resident NK cells
PBMC: peripheral blood mononuclear cells
cNK: conventional NK cells
BM: bone marrow
SLT: secondary lymphoid tissues
MALT: mucosal-associated lymphoid tissue
DC: dendritic cell
NKRM: tissue-resident memory-like NK
TBX21: TBox transcription factor 21
FGL2: fibrinogen-like 2;IRF8, interferon regulatory factor 8
NSCLC: non-small cell lung cancer
LUSC: lung squamous cell carcinoma
LUAD: lung adenocarcinoma
TNBC: triple-negative breast cancer
LGGs: low-grade gliomas
HGGs: high-grade gliomas
CSCC: cervical squamous cell carcinoma
pDC: plasmacytoid DCs
CIN: chromosomal instability
AIS: adenocarcinoma *in situ*
MIA: minimally invasive adenocarcinoma
IAC: invasive adenocarcinoma
CTCs: circulating tumor cells
PVR: poliovirus receptor
GAM: glioma-associated macrophage
CAF: cancer-associated fibroblasts
snRNA-seq: single-nucleus RNA sequencing
ICB: immune checkpoint blockade
ICI: immune checkpoint inhibitor
DCB: durable clinical benefit
HGSC: high-grade serous ovarian cancer
siRNA: small interfering RNA
DMC: 2,5-dimethyl-celecoxib
DHA: docosahexaenoic acid
PCLM: pancreatic cancer liver metastasis
αGC: α-galactosylceramide
iNKT: invariant natural killer T cells
RFR: radiofrequency radiation
MWA: microwave ablation
ADCC: antibody-dependent cellular cytotoxicity
HNSCC: head and neck squamous cell carcinoma
RGS1: regulator of G protein signaling 1
PRGs: pyroptosis-related genes
CAR: chimeric antigen receptor
AML: acute myeloid leukemia
